# Limited Specificity of GroEL-Homolog Interactions with Plant Virus Coat Proteins Challenges Models of Vector-Mediated Virus Transmission

**DOI:** 10.3390/microorganisms14102176

**Published:** 2026-09-27

**Authors:** Tesneem Nusayr, Rebecca Creamer

**Affiliations:** 1 Biomedical Sciences, Life Sciences, Texas A&M University-Corpus Christi, Corpus Christi, TX 78412, USA; 2 Entomology Plant Pathology and Weed Sciences, New Mexico State University, Las Cruces, NM 88003, USA; creamer@nmsu.edu

**Keywords:** PCR-capture, BacterioMatch^TM^ system, β-galactosidase unit, bacterial endosymbiont

## Abstract

Plant viruses often depend on insect vectors for transmission, and interactions between viral coat proteins (CPs) and insect endosymbiont-derived chaperonins may facilitate viral persistence within the vector. This study investigated interactions between a GroEL-homolog protein (GroHp) produced by the beet leafhopper endosymbiont *Sulcia muelleri* (SMBLH GroHp) and several plant virus CPs. Using PCR-capture assays, ELISAs, and bacterial-two-hybrid assays, we evaluated binding between SMBLH GroHp and multiple plant viruses and compared these interactions with those of *Escherichia coli* GroEL. Contrary to expectations, SMBLH GroHp did not exhibit strong specificity toward curtovirus CPs and showed weak interactions across multiple viral proteins, while GroEL displayed stronger and broader binding. Structural and biochemical characteristics of CPs, including charge and isoelectric point, were insufficient to predict binding affinity, suggesting additional molecular determinants influence GroHp-CP interactions. Furthermore, GroHp abundance did not differ between viruliferous and aviruliferous insects, indicating that GroHp is constitutively expressed and not induced by viral presence. These findings suggest that GroHp-CP interactions alone are unlikely to fully determine vector specificity or transmission efficiency. Instead, viral transmission likely depends on combined factors involving viral properties, vector physiology, and potentially additional molecular interactions within the vector.

## 1. Introduction

Plant viruses are economically important and can infect a wide range of host plants [1]. One important family of plant viruses, *Geminiviridae*, contains a circular single stranded DNA (ssDNA) genome, encapsidated in two icosahedral particles [1]. It has nine known genera, *Begomovirus*, *Topocuvirus*, *Curtovirus*, *Becurtovirus*, *Turncurtovirus*, *Capulavirus*, *Mastrovirus*, *Grablovirus*, and *Eragrovirus* [2,3,4,5]. Curtoviruses cause curly top disease (CTD), and are only transmitted by the hemipteran insect, beet leafhopper *Circulifer* (*Neoaliturus*) *tenellus* (Baker), in a persistent circulative (nonpropagative) manner [6]. In New Mexico, CTD has caused sporadic economic losses for chile pepper (*Capsicum annuum* L.) that reached up to 50% loss in 2001 [7]. In 2013 beet curly top disease caused the loss of one million tons of tomatoes in California (http://www.Westernfarmpress.com/vegetables/virus-slams-2013-california-tomato-yields) (accessed on 27 June 2017).

Phloem-feeding insects such as leafhoppers, aphids, and whiteflies harbor bacterial endosymbionts. These endosymbionts produce a GroEL-homolog protein (GroHp) [1]. The beet leafhopper vector of curtoviruses was recently demonstrated to contain a GroHp, produced by its primary endosymbiont, *Sulcia muelleri* (SMBLH GroHp) [8]. The *Escherichia coli* GroEL is an HSP60 that assists in the folding of proteins into their three-dimensional structure [9]. This protein, or its homologues, can be found in most bacteria, including endosymbiotic bacteria. The GroHp produced by the endosymbionts of aphids and whiteflies was shown to interact with the virus coat protein (CP), facilitating virus transmission [10,11,12]. The Gram-negative bacteria *Buchnera aphidicola* is an endosymbiont of the green peach aphid, *Myzus persicae*, which transmits the virus, Potato leafroll virus (PLRV, *Luteoviridae*). This endosymbiont produces a GroHp that can interact with the coat protein (CP) of PLRV [13]. Some insects have more than one endosymbiont, each producing their own GroHps, but the virus interacts with only one copy of this protein [12,14]. Whiteflies (*Bemisia tabaci*, Gen) have several different primary (maternally inherited) and secondary (ecosystem-derived) endosymbionts [10,15,16,17,18]. Depending on the biotype of the whitefly, A-biotype, B-biotype, or Q-biotype, and the begomovirus it carries, the interaction with the CP is restricted to a GroHp from only one of those endosymbionts [10,19]. For example, *B. tabaci* B-biotype transmitting Tomato yellow leaf curl virus Israeli strain (TYLCV-Is) harbors the secondary endosymbiont *Hamiltonella* that cannot be found in Q-biotypes which do not transmit TYLCV-Is. The function of GroHp in a virus/vector system could involve not only protecting the virus from degradation or from detection by the immune system, but also virus trafficking through the aphid and whitefly vectors, and preventing virus aggregation [10,12,20]. Geminivirus CP plays important roles in virion formation, systemic and vector transmission and specificity [13,14,21,22,23,24,25,26,27,28,29,30]. The creation of chimeric viruses, by replacing the CP of a non-transmissible geminivirus with that of a transmissible virus, caused the new virus to be transmitted [13,23]. Moreover, point mutations in the CP of the N-terminal of Tomato yellow leaf curl virus (TYLCV-Is, *Geminiviridae*) led to the loss of interaction with GroHp [27], while in Beet curly top virus (BCTV-Mld, *Geminiviridae*) it led to the loss of vector transmission [31].

Although GroHp produced by the endosymbionts of other insect vectors, such as aphids and whiteflies, has been identified and its interaction with viral CP has been investigated, it is unknown if there is any interaction between the GroHp produced by the *Sulcia muelleri* endosymbiont of the beet leafhopper (*Circulifer tenellus*), SMBLH, and the coat protein of curtovirus. This study aimed to determine whether interactions occur between SMBLH GroHp and several plant virus coat proteins using a bacterial-two-hybrid assay [31], ELISA methods [12], and a PCR-capture assay [32]. In addition, the relative amount of GroHp in viruliferous and aviruliferous beet leafhoppers was examined.

## 2. Materials and Methods

### 2.1. Bacterial Strains

The plasmids used for this study were as follows: pGem-T Easy (Promega, Madison, WI, USA), for cloning; pRset system (Invitrogen, Waltham, MA, USA) and the IMPACT kit TM (New England Biolab, Ipswich, MA, USA), for protein expression; and pBT and pTrg plasmids from BacterioMatch (Stratagene, Santa Clara, CA, USA), for the bacterial-two-hybrid assay.

### 2.2. Sources of Virus, Leafhoppers, Infected Plants, and Antibodies

The beet leafhoppers carrying the curtovirus BCTV-Svr strain used in this study were gifts from Carl Strausbaugh, USDA, Kimberly, ID, while the BCTV-PeCTV strain was collected from local infected fields at Las Cruces, NM, USA. Both strains of curtovirus cause Beet curly top disease, and are vectored by the beet leafhopper. Beet leafhoppers were reared on sugar beet plants maintained at 28 °C day/26 °C night with a 16 h photoperiod. Seeds of sugar beet, Beta vulgaris, breeding line P1518-6, were provided by Kelly Richardson, USDA, Salinas, CA, USA. Curtovirus (BCTV-Svr, and BCTV-PeCT)-infected chile pepper plants were collected from Leyendecker Plant Science Research Center, Las Cruces, NM, USA in May 2015, and stored at −20 °C. Tobacco mosaic virus (TMV)-infected tobacco leaves were obtained from SONOMA cigarettes, Commonwealth Brands, Inc. (Bowling Green, KY, USA) Mfg. Reidsville, NC 27320. Plant homogenates were prepared by crushing frozen symptomatic infected or uninfected leaves (~0.1 g) into powder and adding 2 mL TBST (10 mM Tris-HCl pH 7.6, 150 mM NaCl, 0.05% Tween 20), or ELISA coating buffer (15 mM Na_2_CO_3_, 35 mM NaHCO_3_, and 0.02% NaN_3_), as the assay requires. The preparation was gently mixed for at least 30 min, at 4 °C, and centrifuged at 7000× *g* for 5 min. The supernatant homogenate was stored at 4 °C and used within seven days. The TYLCV-infected plants and the noninfected plants were prepared similarly. TYLCV were prepared at Dr. Jane Polston’s lab, University of Florida. The antibodies used in this study included anti-*E. coli* GroEL-antibody (Sigma, St. Louis, MO, USA, G6532), diluted in TBST (1:10,000), and anti-BCTV-CP-antibody, diluted 1:200 in TBST, as described in [33]. A goat anti-rabbit IgG (whole molecule)–alkaline phosphatase antibody produced in goat (Sigma, A0418) was used as a secondary antibody. The anti-BCTV-CP-antibody had a stock concentration of 1 mg/mL. Two plasmid constructs were used as CP templates: Begomovirus Bean golden yellow mosaic virus (BGYMV) segment A, GenBank: M91604.1 (gift from Dr. Steve Hanson, NMSU); and a pUC119 construct containing the closterovirus Beet yellows virus ORF # 5 p24 minor coat protein (BYV-CPm), GeneBank: U71437.1. The pBT-Zein plasmid (10 kDa maize protein, GeneBank: X07535.1) construct was a gift from Dr. Jennifer Randall, NMSU.

### 2.3. Cloning, Over-Expressing and Purification of GroHp and E. coli GroEL

Full-length SMBLH GroHp and *E. coli* GroEL were amplified and cloned into expression vectors, and purified, as described in [8], and the integrity of the purified proteins was confirmed by electron microscopy by the same group. The purified products were stored at −80 °C and used within 10 days of preparation.

### 2.4. Cloning SMBLH groHp and E. coli groEL Genes into the BacteriMatch^TM^ System

The primers used for amplifying GroHp and CP genes with their corresponding plasmid and the restriction site/enzyme used for cloning are listed in Table S1. A standard PCR reaction with standard buffers was performed with the appropriate DNA template. The PCR cycles were as follows: initial denaturation at 94 °C for 5 min, 35 cycles of 94 °C for 1 min, annealing cycle with various temperatures (Table S1) for 2 min, and 72 °C for 1.5 min, and a final extension at 72 °C for 10 min, then ending at 4 °C. PCR amplicons were electrophoresed in a 1% agarose gel in 1X TAE buffer (40 mM Tris-acetate and 1 mM EDTA), stained by GreenView^TM^ (ABP Biosciences, Beltsville, MD, USA). Freshly amplified full lengths of SMBLH *groHp* and *E. coli groEL*, BCTV-PeCT-CP, BGYMV-CP, and BYV-CPm were purified using a QIAquick PCR Purification Kit (QIAGEN, Hilden, Germany), according to the manufacturer’s instructions. The amplicons were cloned into pGEM-T Easy according to the manufacturer’s instructions. The cloned and transformed plasmids were propagated in 5 mL of LB broth supplemented with 100 µL/mL ampicillin. The plasmids were purified using E.Z.N.A. Plasmid DNA Mini Kit I (OMEGA) (OMEGA Bio-Tek, Norcross, GA, USA), then digested with suitable enzymes. The digested products were separated on a 1% agarose gel, then purified using the QIAquick gel Purification Kit (QIAGEN, Hilden, Germany). Both *groEL and groHp* genes were cloned into pBT at the *Not*I and *Xho*I sites, and the coat proteins were cloned into pTrg at the *Bam*HI and *Xho*I sites. All cloned constructs were propagated in BacterioMatch^TM^ reporter cells supplemented with chloramphenicol (30 µg/mL) for pBT, and tetracycline (15 µg/mL) for pTrg. The integrity of the cloned genes was confirmed by sequencing (MCLAB, South San Francisco, CA, USA).

### 2.5. ELISA Screening for Interaction of Anti-E. coli GroEl Antibody with Both SMBLH GroHp and E. coli GroEL

Purified GroEL and GroHp proteins were diluted in ELISA coating buffer. The stock concentration of proteins was 1 mg/mL. A series of 1:10 serial dilutions (from 10^−1^ to 10^−8^) was prepared, and 100 µL of each dilution was added to separate wells in the columns of an ELISA plate. The plate was incubated at room temperature for 3.5 h on a tilting platform to ensure uniform coating. Following incubation, the wells were washed three times with TBST wash buffer and then blocked with 200 µL of 5% non-fat dry milk in TBST to reduce nonspecific binding. After blocking for 1.5 h at room temperature, the wells were washed three times with TBST. The primary antibody, a polyclonal anti-*E. coli* GroEL antibody diluted 1:10,000 in TBST, was added to each well and incubated for 3 h at room temperature. After another series of three TBST washes, the wells were incubated overnight at 4 °C with a secondary antibody, 1 mg/ml purified IgG; alkaline phosphatase–conjugated goat anti-rabbit (Sigma, St. Louis, MO, USA) was used at 1:2000 dilution. Absorbance at 405 nm was determined using an Emax plate reader (Molecular Devices, Sunnyvale, CA, USA). The experiment was repeated twice to ensure reproducibility. Data analysis was performed using Microsoft Excel and an online ANOVA with *t*-test.

### 2.6. Preparation of BLH Body Parts

Viruliferous and aviruliferous beet leafhoppers (BLHs) were dissected into parts: head, thorax and abdomen, or head and thorax/abdomen; each part was crushed vigorously using sterile glass beads in Eppendorf tubes with a plastic pestle and homogenized in the appropriate assay buffer.

### 2.7. Western Blot Detection of BCTV-CP in Viruliferous BLHs

Fiveviruliferous BLHs were dissected into the three body groups, and another three were kept whole. The samples were homogenized in 50 µL sample buffer and boiled for 10 min. The crude protein extracts were subjected to 10% SDS-PAGE, at 100 V, followed by Western blot. The membrane was blotted with 5% non-fat milk, washed, then incubated with anti-BCTV-CP-Ab, as a primary antibody.

### 2.8. ELISA Detection of GroHp in the Head, Thorax, and Abdomen of Viruliferous and Aviruliferous BLH

The head, thorax, and abdomens from ten viruliferous and aviruliferous BLHs were homogenized in ELISA coating buffer (10 BLH part/100 µL). From each preparation, 100 µL was used to coat three replicate wells per body part. In addition, two columns of the ELISA plate were coated with purified GroHp protein at a concentration of 1 µg/mL in coating buffer, and another column was left uncoated to serve as a background control. After sample application, the plates were incubated overnight at 4 °C, followed by three washes with TPBT. Blocking was performed at room temperature for 1 h using TBST supplemented with 2% BSA, after which the plates were washed three times with TBST. The primary antibody, anti-*E. coli* GroEL (diluted 1:10,000), was added and incubated at 37 °C for 1.5 h. Following another set of three TBST washes, the secondary antibody (diluted 1:2000) was applied and incubated for 3 h at room temperature. The plates were then washed, and signal detection was carried out. The experiment was repeated twice to ensure reproducibility. Data analysis was performed using Microsoft Excel and an online ANOVA with *t*-test.

### 2.9. Confocal Microscope Detection of GroHp and BCTV in Head, Thorax, and Abdomen of BLH

The head, thorax, and abdomen of viruliferous BLHs were prepared in sterile TBST, then with NH_4_Cl plus glycine. The cell membranes were incubated in PBS containing 1% Triton X-100 overnight and then blocked with 10% normal horse serum. The samples were incubated with either anti-*E. coli* GroEL-antibody (10,000 dilution), or with anti-BCTV-CP-antibody (1:50 dilution) for 2.5 h, washed again, then incubated for 2.5 h with diluted (10 µL/mL TBST) Alexa Flur^®^ 647 goat anti-rabbit SFX IgG (Invitrogen, Eugene, OR, USA). The samples were washed again and viewed using confocal microscopy (Carl Zeiss, Oberkochen, Germany). The control was BLH body segments prepared the same way but without using a primary antibody. The red fluorescently labeled samples were visualized with a model TCS SP5 laser scanning confocal microscope (Leica Microsystems, Mannheim, Germany). The red nanophores were excited with 488 Argon laser at 500–550 nm range (Carl Zeiss, Oberkochen, Germany), and the HeNe laser PMT2 filter gave the maximum excitation at line 632 (Carl Zeiss, Oberkochen, Germany).

### 2.10. Western Blot Detection of GroHp in BLH

The head, thorax, and abdomen of six viruliferous BLHs were crushed in 100 µL of sample buffer (0.125 M Tris-HCL, pH 8.0, 20% glycerol, 1% β-mercaptoethanol, 0.001% bromophenol blue), then boiled for 10 min. The samples were subjected to 10% SDS-PAGE, at 100 V, followed by Western blot. The membranes were blocked with 5% non-fat milk, washed, then incubated with either anti-*E. coli* GroEL-antibody, or anti-BCTV-CP-antibody.

### 2.11. PCR Detection of BCTV in BLH

Five viruliferous BLHs were dissected into two parts: heads and thorax/abdomen. The two body parts were homogenized in PBS buffer. Curtovirus was detected by PCR using BCTV-PeCT-CP primers.

### 2.12. Western Blot Detection of BCTV-CP Interaction with GroHp/GroEL

One ml of LB medium was inoculated with one colony of *Escherichia coli* (MG1655) and incubated at 42 °C, for 13 h, to over-induce the production of GroEL. The culture was centrifuged for one minute at 13,000× *g*. The supernatant was discarded, and the pellet was resuspended in 500 µL of sample buffer then boiled for 10 min. Crude protein from BLHs was prepared by homogenizing five adults in sample buffer, and boiling for 10 min. The *E. coli* sample and BLH crude protein were subjected to 10% SDS-PAGE, at 100 V, followed by Western blot. The affinity of GroHp and GroEL to BCTV-infected plants was executed as in [20]. Briefly, the blotted membrane was blocked with 5% non-fat milk, washed, then incubated with BCTV-Svr-infected plant homogenate, washed, then incubated with anti-BCTV-CP-Ab primary antibody.

### 2.13. PCR-Capture of BCTV-PeCTV and TYLCV GroEL/GroHp-Coated Tubes

As described previously [34], 100 µL of partially purified SMBLH GroHp and *E. coli* GroEL diluted in ELISA coating buffer were added to PCR tubes. The starting concentration was 1 mg/mL and serial dilutions were prepared starting with 0.1 mg/mL dilution and ending with 0.01 ng/mL. The tubes were incubated at 37 °C for 3 h, then washed with TBST and blocked for 1.5 h. In total, 50 ul of homogenates of infected plants (BCTV-PeCTV, and TYLCV) was added to the tubes, incubated overnight at 4 °C, then washed. PCR master mix, with primers for the BCTV-PeCT-CP gene (F’: 5′-ATGTCGAGATTTACAAAAGGTA-3′, R’: 5′-CAATCTTCAATCTCG-3′; annealing temp: 50 °C) and primers for TYLCV-CP (F1: 5′-GGGCCTAGAGACCTGGCCCAC-3′, and Reverse’: 5′-CCGGTAATATTATACGGATGGC-3′; annealing temp: 57 °C) were added to the tubes. The negative control was plant homogenate added to non-coated tubes. Two positive controls were used: DNA extracted from the infected plants, and PCR tubes coated with infected plant homogenate in coating buffer.

### 2.14. PCR-Capture of TMV Using GroEL/GroHp-Coated Tubes

The PCR-capture assay was repeated but with TMV-infected plants. After incubating the coated and blocked tubes with TMV-infected plant homogenate and subsequent washes, the presence of TMV was tested using a ProtoScript^®^ II First Strand cDNA Synthesis kit (New England Biolabs # E6560L, New England Biolabs, Ipswich, MA, USA), following the manufacturer’s instruction. TMV presence was detected using TMV primers (F’: 5′-ATGTCTTACAGTATCACTACTCC-3′, R’: 5′-TCAAGTTGCAGGACCAGAGGT-3′).

### 2.15. ELISA Detection of SMBLH GroHp and E. coli GroEL Interaction with Infected Plants

Plant homogenates were prepared in ELISA coating buffer. Extracts from plants infected with TMV, BCTV, and TYLCV were used to coat separate rows of ELISA plates. The control wells were coated with purified GroHp or GroEL proteins. The plates were incubated for 3 h at room temperature, then washed three times with TBST. The wells were subsequently blocked using TBST containing 2% BSA and incubated for 1.5 h at room temperature. After blocking, the plates were washed again and overlaid with serial dilutions of purified GroHp and GroEL proteins, 0.1 mg/mL to 0.01 ng/mL in TBST containing 2% BSA, and incubated overnight at 4 °C. The plates were washed three times with TBST containing 2% BSA. The plate was incubated with primary antibody, anti-*E. coli* GroEL, then was washed; the plate was incubated with a secondary antibody (diluted 1:2000) for 3 h at room temperature. Following a final set of washes, the plates were developed for signal detection. This assay was performed twice. The OD of the controls were considered 100% interaction and were used to calculate the interaction percentage for the plant extract.

### 2.16. ELISA Detection of SMBLH GroHp and E. coli GroEL Interaction with Uninfected Plants

The same procedure as above was repeated with the homogenates uninfected tomato, sugar beet, and pin oak leaves, by preparing them in ELISA coating buffer and then using them to coat the columns of ELISA plates. The plates were incubated for 3 h at room temperature, then washed three times with TBST. The wells were blocked using TBST containing 2% BSA and incubated for 1.5 h at room temperature. After another wash, serial dilutions of purified GroHp and GroEL proteins (0.1 mg/mL to 0.01 ng/mL) were prepared in TBST with 2% BSA. One column was coated in the same serial dilution with GroHp, and another with GroEL. The plates were incubated overnight at 4 °C and then washed three times with TBST containing 2% BSA. The primary antibody, anti-*E. coli* GroEL (diluted 1:10,000 in TBST), was added and incubated for 1.5 h at 37 °C. Following another set of three washes with TBST, the secondary antibody (diluted 1:2000) was added and incubated for 3 h at room temperature. The plates were then washed and developed for signal detection. Controls were as mentioned above.

### 2.17. Analysis of GroEL/GroHp-CP Interactions in the BacterioMatch^TM^ Two-Hybrid System

Fourteen pairs of the cloned pBT and pTrg constructs were co-transformed into the BacterioMatch^TM^ *E. coli* reporter strain for interaction analysis (Table 1). The genes for GroHp, GroEL, and the coding region of the maize protein, Zein, were cloned into the “bait” pBT vector, while the genes for the three viruses’ CP were cloned into the “prey” pTrg vector. The co-transformants were plated on LB plates containing 250 µg/mL carbenicillin, 15 µg/mL tetracycline, 30 µg/mL chloramphenicol, and 50 µg/mL kanamycin. Colonies which grew on these plates were then tested for β-galactosidase activity.

#### Beta-Galactosidase Assays

The β-galactosidase assay was performed as described by [31]. The β-galactosidase activity was determined by calculating the Millers Units.

### 2.18. Statistical Analyses

One-way and two-way ANOVAs (Analyses of Variance) were conducted to assess whether differences in β-galactosidase activity and ELISA measurements among the sample means were due to random variation or reflected systematic differences. All statistical analyses were performed using a significance level of α = 0.05. The null hypothesis (H_0_) was rejected when the calculated F-value exceeded the critical F-value, indicating significant variation among group means. To identify which specific group comparisons were statistically significant, additional post hoc analyses were performed using Student’s *t*-test for independent, unpaired, two-tailed comparisons, as well as Tukey’s test for multiple group comparisons. For both tests, the null hypothesis was rejected when the calculated t-value exceeded the critical t-value.

### 2.19. Coat Protein Analysis

The online tool ProtCal (http://protcalc.sourceforge.net/) was used to calculate the charge, isoelectric point (pI), and arginine count of the CPs analyzed in this study.

## 3. Results

### 3.1. Cross-Reactivity of Anti-E. coli GroEl Antibody with SMBLH GroHp and E. coli GroEL

To determine whether there is cross-reactivity of anti-*E. coli* GroEL antibody with SMBLH GroHp and *E. coli* GroEL proteins in subsequent experiments, an ELISA was conducted. A one-way ANOVA revealed a significant difference in the interaction of anti-*E. coli* GroEL antibody with SMBLH GroHp and *E. coli* GroEL proteins (Figure 1, Table S2). The differences were consistent across equivalent dilutions. The anti-*E.coli* GroEL antibody had about 1.12 times more affinity with GroEL than GroHp. Therefore, the subsequent interaction ELISAs included purified GroHp and GroEL controls, and the signal for each test sample was expressed relative to the corresponding purified protein control.

### 3.2. Detection of GroHp and BCTV in the BLH Body Regions

To determine whether GroHp and BCTV occurred in the same regions of viruliferous BLHs, insects were disected into head, thorax, and abdomen samples and examined by confocal microscopy using either anti-*E. coli* GroEL antibody or the anti-BCTV coat protein antibody. GroHp-associated fluorescence was distributed throughout the head, whereas BCTV-Svr-associated fluorescence was localized primarily to the salivary glands (Figure S1). Detection of either target in the thorax and abdomen by confocal microscopy was inconclusive.

The same body regions were also examined by Western blotting and PCR. Western blot probed with the anti-*E. coli* GroEL antibody contained bands of the size of GroHp (~60 kDa), in the head, thorax, and abdomen of BLH (Figure S2). A crude protein preparation from a whole beetle was used as a control which produced a faint band corresponding to the size of GroHp. In contrast, Western blotting was not sensitive enough to detect BCTV-Svr in any of the BLH body parts. PCR analysis of DNA from the head and the combined thorax/abdomen samples produced the expected approximately 300 bp BCTV amplicon, the size of Rep primers, in both preparations, confirming the presence of viral DNA in both body regions (Figure 2).

### 3.3. Comparison of GroHp Abundance in Viruliferous and Aviruliferous BLHs

To test whether BCTV infection status affected GroHp abundance, heads, thoraxes, and abdomens from viruliferous and aviruliferous BLHs were analyzed by ELISA. For each infection-status group, homogenates prepared from ten corresponding body parts were applied to three replicate wells and detected with the anti-*E. coli* GroEL antibody; purified GroHp-coated wells and uncoated wells served as positive and background controls, respectively. Two-way comparisons detected no significant effect of infection status, body region, or the interaction between these factors on the GroHp signal (Table S3). Thus, GroHp levels did not differ significantly between viruliferous and aviruliferous insects or among the head, thorax, and abdomen within either group.

### 3.4. BCTV-Svr Interacts with GroHp and GroEL in Crude Protein Preparations

To test whether BCTV-Svr in infected plant homogenate could interact with GroHp or GroEL in crude protein preparations, proteins from whole BLHs and heat-shock-induced *E. coli* were separated by SDS-PAGE and transferred to membranes. The membranes were incubated with BCTV-Svr-infected plant homogenate, washed, and then probed with the anti-BCTV coat protein antibody to detect the virus retained at the positions of the blotted proteins. Both the BLH and *E. coli* preparations produced three immunoreactive bands with similar migration patterns (Figure 3). In each preparation, two bands migrated between approximately 45 and 60 kDa; a third, lower-molecular-mass band was prominent in the *E. coli* preparation and faint in the BLH preparation. The corresponding control containing BCTV-Svr-infected plant material without BLH or *E. coli* protein did not produce these bands (Figure 3) [12,32].

### 3.5. ELISA Comparison of SMBLH GroHp and E. coli GroEL Binding to Infected and Uninfected Plant Homogenates

Comparisons of the binding of purified GroHp and GroEL proteins with homogenates from BCTV-infected sugar beet, TYLCV-infected tomato, and TMV-infected tobacco, together with the corresponding uninfected plant homogenates when available, were assessed using ELISA. Serial dilutions of purified GroHp or GroEL were added, and retained protein was detected with the anti-*E. coli* GroEL antibody. Purified GroHp- and GroEL-coated wells were included on each plate, and the absorbance of each plant-homogenate sample was expressed as a percentage of the signal from the corresponding purified protein control. GroHp produced uniformly low signals with the both virus-infected homogenates: approximately 4.5% with BCTV-infected sugar beet and TYLCV-infected tomato (Figure 4). GroHp also produced a low signal with uninfected sugar beet (approximately 4–6%) but a higher signal with uninfected tomato (approximately 10–12%) (Figure 4; Tables S4 and S5).

Direct comparison of GroEL and GroHp showed that GroEL was retained more strongly than GroHp by both infected and uninfected plant homogenates. For sugar beet, the GroHp signals did not differ significantly between BCTV-infected (approximately 4%) and uninfected (approximately 6%) homogenates, whereas the corresponding GroEL signals were approximately 22% and 14%, respectively (Figure 5; Table S6). For tomato, GroEL produced a signal of approximately 20% with TYLCV-infected homogenate and approximately 48% with uninfected homogenate (Figure 6). The difference between the infected and uninfected tomato samples was significant. The largest difference between GroHp and GroEL was observed with TMV-infected tobacco homogenate: the GroEL signal was approximately 49%, compared with approximately 4.5% for GroHp, an approximately 11-fold difference (Figure 7; *p* < 0.01).

To determine whether binding was specific to virus-infected material, GroHp and GroEL were also tested against homogenates of uninfected pin oak, sugar beet, and tomato. GroHp signals differed significantly among the three uninfected homogenates and were highest for pin oak (approximately 39%), followed by tomato (approximately 12%) and sugar beet (approximately 6%) (Figure 8; Table S4). GroEL showed the same rank order but higher signals: approximately 83% for pin oak, 47% for tomato, and 16% for sugar beet (Figure 9; Table S4).

**Figure 7:**
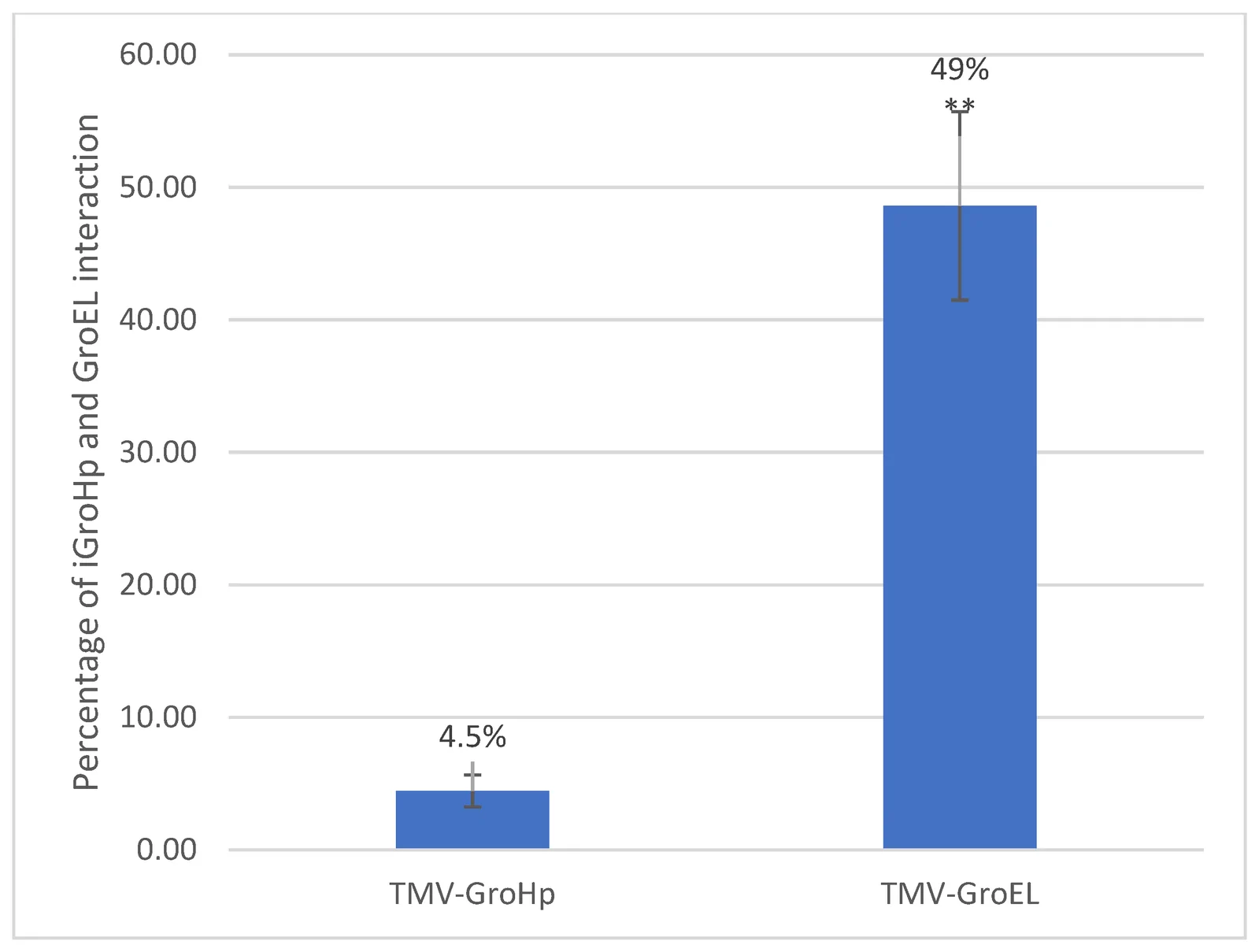
The percentage of GroHp vs. GroEL interaction with TMV-infected tobacco. An ELISA was done to determine if SMBLH GroHp and *E. coli* GroEL interact with TMV-infected tobacco leaf extract. The results show that there was significant interaction between GroHP and TMV-infected plants (~4.5%) and GroEL and TMV-infected plants (~49%). GroEL has more affinity with TMV-infected plants of about 11 times greater than GroHp. The controls were coating ELISA wells with purified protein, one row with GroHp and another with GroEL, instead of TMV-infected tobacco plant homogenate, then adding GroHp protein to the GroHp-coated wells and GroEL to the GroEL-coated wells. The O.D. readings were used to calculate the percentage of protein interactions. (**) is *p*-value < 0.01.

### 3.6. Purified GroHp and GroEL Capture BCTV, TMV, and TYLCV from Infected Plant Homogenates

The PCR-capture assay tested whether purified GroHp and GroEL could retain virus particles from infected plant homogenates after repeated washing. PCR tubes were coated with serial dilutions of GroHp or GroEL (0.1 mg/mL to 0.01 ng/mL); blocked; incubated with BCTV-PeCT-, TYLCV-, or TMV-infected plant homogenate; and washed. Captured BCTV-PeCT and TYLCV were detected by CP-specific PCR, whereas captured TMV was detected by reverse transcription followed by CP-specific PCR. Uncoated tubes served as negative controls, and extracted nucleic acid and infected-plant-homogenate-coated tubes served as positive controls. Virus-specific amplicons were obtained from both GroHp- and GroEL-coated tubes for all three viruses at every coating concentration tested, including 0.01 ng/mL; no amplicons were detected in the uncoated controls or after incubation with uninfected plant homogenate (Table 2; Figure S3). Thus, the assay demonstrated capture by both proteins but did not distinguish differences in binding strength among the three viruses.

### 3.7. Physiochemical Analysis of the Viral Coat Proteins

Coat protein for several viruses was analyzed using the online tool ProtCal to assess their binding with GroHP and GroEL. The BYVs-CPm had a low charge at pH 7 (1.6), Pi of 7.84, and 6.0% Arg residues, as well as being isometric-shaped. The BGYMV-CP has a charge of 24.4, Pi of 9.88, and 8.4% Arg residues, as well as being geminate in shape, while BCTV-PeCT-CP had a charge of 9.99, Pi of 22.6, and 7.1% Arg residues, and a geminate shape. The TYLCV-CP had a pH of 7.4, Pi 6.5, 0.68% Arg, a negative charge and geminate shape. The TMV-CP is filamentous with a Pi of 4.59, 6.9% Arg, pH 7.4, and a negative charge.

### 3.8. Purified SMBLH GroHp Binds BCTV-PeCTV-CP More Strongly than Other CPs

The BacterioMatch™ two-hybrid assay was used to compare pairwise interactions between three test proteins and three viral coat proteins. SMBLH GroHp, *E. coli* GroEL, or maize zein was expressed as the pBT bait fusion, and BCTV-PeCT CP, BGYMV CP, or BYV-CPm was expressed as the pTrg prey fusion. The growth of co-transformants on selective medium indicated interaction between the bait and prey proteins (Table 1). All nine test pairs produced colonies, whereas pairs containing an empty bait or prey vector did not.

The units of β-galactosidase produced by the positive controls provided by the system (Gal 4 and Galp 11) were considered to be 100% affinity. Table 3 shows the construct co-transformants of pTrg + pBT, with the percentages of β-galactosidase unit, compared to the positive controls.

A one-way ANOVA showed significant variation among the nine test pairs (F = 39.46, exceeding the critical F value of 2.07; Table S7). Pairwise Student’s *t*-tests were then used to determine which interactions differed significantly. The β-galactosidase values and statistical groupings are presented in Table 3 and Figure 10.

The two strongest interactions were SMBLH GroHp with BCTV-PeCT CP (~41%) and *E. coli* GroEL with BYV-CPm (42%); these two pairs were significantly stronger than the remaining test pairs (Figure 10; *p* < 0.05). SMBLH GroHp interacted less strongly with BGYMV CP (~29%) and BYV-CPm (31%) than with BCTV-PeCT CP. *E. coli* GroEL produced similar interaction values with BCTV-PeCT CP (~29%) and BGYMV CP (~27%), but a higher value with BYV-CPm (42%). The non-chaperone zein control also interacted with all three coat proteins, with values of 34% for BCTV-PeCT CP, 26% for BGYMV CP, and 29% for BYV-CPm.

## 4. Discussion

Plant viruses avoid degradation during their transit through the insect vector, ensuring transmission. A factor that might protect the virus is the interaction between a GroEL-homolog protein (GroHp), produced from the insect’s endosymbiont, and the CP of the virus [11,12,20,28]. The GroHp from the beet leafhopper (BLH) *C. tenellus* endosymbiont had been previously identified as the new protein from a new strain named *Ca. S. muelleri* SMBLH [8]. This work compares GroHp interaction with different plant virus CPs and examines if there is a difference between the amount GroHp of viruliferous and aviruliferous BLHs.

The interaction between SMBLH GroHp and curtovirus CP had not been previously explored, nor between GroHp and other plant virus CPs. Interactions between endosymbiont GroHps and viruses (CPs) are more likely when certain conditions are met [34]. The interaction between whitefly endosymbiont’s GroHp and virus CP appears to be favorable when the CP has a positive charge (>7.2), a high isoelectric point (Pi > 9.0), richness in arginine, and preferably a globular (or geminate) shape [34]. Binding assays of whitefly GroHp and different plant viruses not transmitted by whiteflies, which had filamentous shapes and/or low or negative charges, low Pi, and low arginine percentage, showed failure of binding [34]. These conditions do not appear significant for GroHp interaction with infected plant homogenates. However, there is a preference for GroHp interaction with purified CPs during the bacterial-two-hybrid assay, which could be due to the more sensitive nature of such an assay compared to the others, or it could be a plant component that interferes with the action of GroHp that does not exist in the vector.

The PCR-capture assay aimed to examine the interaction of purified GroHp to BCTV-PeCT-infected plant homogenate and compare it to that of TYLCV- and TMV-infected plant homogenate and determine how that differed from *E. coli* GroEL binding to the same viruses. GroHp- and GroEL-coated PCR tubes bound to BCTV-PeCT-, TYLCV-, and TMV-infected plant homogenates. The *E. coli* GroEL was predicted to interact with BCTV-PeCT, TYLCV, and TMV, while SMBLH GroHp would favor interacting with BCTV-PeCT. The PCR-capture assay showed that both GroHp and GroEL showed no difference in interaction with the three viruses. The virus particles bound at all concentrations of GroHp/GroEL (0.1 mg/mL–0.01 ng/mL). The PCR-capture assay only investigates the interaction between the proteins but could not detect any differences between the bindings. We expected TYLCV to show more specificity [10], and not interact with GroHp, so it was surprising that both GroHp and GroEL showed interaction with all infected plant homogenates. The lack of differentiation in the PCR-capture assay suggests that this method detects general binding but may lack the sensitivity to distinguish differences in binding strength or specificity. Therefore, additional assays were necessary to evaluate interaction specificity.

Further interaction assays were conducted using ELISA. We assayed to determine if the affinity of *E. coli* anti-GroEL antibodies to SMBLH GroHp is comparable to *E. coli* GroEL. Our results showed that the anti-GroEL antibody’s affinity to *E. coli* GroEL is about 1.12 times more than to SMBLH GroHp. To minimize any effect of such difference in affinity we always ran a purified SMBLH GroHp and purified *E. coli* GroEL every time we did an ELSA interaction assay and calculated the percentage of interaction against the purified proteins and anti-GroEL antibody.

Using ELISA to compare GroHp and GroEL interaction between infected and uninfected plant homogenates yielded unexpected results. SMBLH GroHp showed consistently lower interaction levels across infected plant samples compared with *E. coli* GroEL. Although GroHp exhibited some interaction with viral proteins, the interaction was not as strong or as selective as expected. The SMBLH GroHp exhibited very low interaction with TMV-infected tobacco (4.5%). This could be because TMV does not meet the conditions required to interact with GroHp as explained in [34].

Interestingly, GroHp had the same low affinity with sugar beet homogenates. This becomes more puzzling when the interaction between GroHp and uninfected sugar beet shows the same low affinity (~4%); in fact, there is no significant difference between BCTV-infected and uninfected sugar beet when their homogenates are incubated with GroHp. Sugar beet is a natural host of curtovirus [6,35] and may contain unknown components in its homogenate that promote GroHp degradation, potentially reducing GroHp-mediated interference with virus infection. The GroHp interaction with TYLCV-infected tomatoes was ~4%, similar to GroHp with BCTV-infected and uninfected sugar beet homogenate. GroHp interaction with homogenate from uninfected tomato homogenates was significantly more (~10%) which reinforces that the interaction with the CPs is nonspecific.

When compared to GroHp, *E. coli* GroEL showed substantially higher overall interaction with TMV-, BCTV-, and TYLCV-infected plant samples (~49%, ~22% and ~20%, respectively), suggesting a stronger affinity or broader cross-reactivity. GroEL interacted more strongly with uninfected tomato plants (~48%) than with TYLCV-infected tomatoes (~20%). Furthermore, GroEL had a significant interaction with uninfected sugar beet and uninfected pin oak plant homogenates (~48%, ~13%, and ~82%, respectively). This was not surprising as GroEL has been called promiscuous because it can interact with many other proteins [36], and GroEL can be used to offer resistance against TYLCV [32,37]. These results suggest that while GroHp and GroEL share structural and functional similarities, their interaction behaviors with plant virus CPs may differ in both specificity and affinity. TYLCV-CP shows specificity in binding with the GroHp produced from the secondary endosymbiont from its own vector, the whiteflies (*Bemisia tabaci*, Gen) [10,12,19,20].

Our bacterial-two-hybrid assay investigated the binding affinity of SMBLH GroHp to three different CPs, BCTV-PeCT, BGYMV, and BYV, which was then compared to the binding affinities of *E. coli* GroEL and Zein protein to the same CPs. Any SMBLH GroHp affinity, or binding, to BYV-CPm was expected to be very low, so it was surprising to find that GroHp can bind to BYV-CPm, at a similar affinity to its binding to BGYMV-CP, though BYV-CPm does not have the favorable conditions for binding mentioned above [34]. The BYV-CPm plays a role in virus encapsidation. It shows affinity with *E. coli* GroEL, similar to that of GroHp to BCTV-PeCT-CP. This could be explained as BYV encode and interact with two virion proteins, HSP70 homolog (HSP70h), and p61. These two possible chaperones are important for virion assembly [38]. Heterologous CPm (minor coat protein) can restore encapsidation, in the presence of HSP70h, and p61 [38]. The strong affinity between *E. coli* GroEL and BYV-CPm might be due to the virus utilizing the ATPase domain of the GroEL itself [38]. Interestingly, *E. coli* GroEL affinities to both BCTV-PECTV-CP and BGYMV-CP were similar (~29% and ~27%, respectively). Furthermore, the binding affinity of GroHp to BCTV-PeCT-CP was greater than with TYLCV-CP. The difference in binding was not very large (~41%, and ~29%, respectively). These observations indicate that structural or physicochemical properties alone are insufficient to predict binding behavior and that additional molecular features may influence GroHp-CP interactions. The maize Zein protein was used to investigate the binding of CPs to a non-chaperone protein. All three CPs were predicted to have similar affinities in binding to Zein; however there was a difference in the affinity in binding to BCTV-PeCT-CP, compared to BGYMV-CP, while binding to BYV-CPm was intermediate. This could be because an unknown agent of the CP itself determines its binding preferences.

The interaction between GroHp and virus CP had been investigated for other viruses/insect combination. Purified GroHp from vector and non-vector aphids, *M. persica*, *Acyrthosiphon pisum*, *Rhopalosiphum padi*, and the *E. coli* GroEL, could interact at different affinities with six luteoviruses, as well as the luteovirus-like virus, Pea enation mosaic virus (PEMV) [12]. Mutated GroHp and virus CP can lead to loss of interaction, and transmission. SMBLH GroHp possible specificity in interacting to BCTV-CP had not been investigated. Mutation assays of the CP have focused on its CP-CP, and CP-GroHp interactions [27], infectivity, vector uptake and transmission, and virion formation [23,28,39,40,41]. Few studies have been conducted about CP interaction with other chaperone proteins [14,38,42,43], and none about the CP interaction with other non-chaperone proteins. The results of this study suggest that the virus CP controls the binding with the BLH endosymbiont GroHp. GroHp binds BCTV-PeCT-CP significantly better than the other two CPs, but was lower than expected. This points to other factors, perhaps from the insect vector that mediated this interaction, and thus, transmission.

Some of the methods used in this study have limitations associated with the assays: Western blotting involves proteins that have been denatured during SDS-PAGE and subsequently immobilized on a membrane, where they may only partially renature and therefore may bind differently from proteins in their native conformation; PCR-capture has an inherent problem that not all proteins will bind equally to the PCR tubes; and all serological assays have the potential of nonspecific binding. Furthermore, the stability of the proteins could be a concern in ELISA due to its lengthy process.

The localization of curtovirus and GroHp within the BLH had been investigated by Alkhatib et al. [38] who found curtovirus-BCTV particles mainly in the anterior midgut and some in the salivary principle glands, while GroHp was detected in the salivary glands and in the different parts of the midgut. We were able to visualize both GroHp and BCTV-CP in the head, using confocal microscopy. GroHp appeared to be distributed all over the head while the virus was localized to the salivary glands.

We detected GroHp in the head, thorax, and abdomen of BLH using Western blot assay but failed to detect curtovirus. This may be due to low virus titer or poor curtovirus antibody sensitivity. However, PCR could detect BCTV in the salivary glands, hemolymph, and digestive tract of the BLH [39], and we were able to detect BCTV-PeCTV in the head and thorax/abdomen body parts of viruliferous BLH. However, no significant difference in GroHp abundance was observed between viruliferous and aviruliferous insects. This suggests that GroHp might be constitutively expressed in the vector and that its abundance alone is unlikely to be directly regulated by viral infection status. Instead, GroHp may function as a readily available chaperone within the vector, capable of interacting with viral proteins when present.

The presence of both BCTV and GroHp in the same vector body parts might lead to interaction, but GroHp does not exhibit the strong specificity observed in other insect–virus systems [10,13,14,15,16,17,18]. The results indicate that GroHp-CP interactions alone may not fully determine vector specificity or transmission efficiency. Instead, virus transmission likely depends on a combination of factors, including CP structure, vector biology, GroHp availability, and additional molecular interactions within the insect. The GroHp-CP interaction could protect the virus from degradation while circulating in the hemolymph as with begomovirus (family *Geminiviridae*) and their whitefly vectors [10,12,19]. This interaction might be passive, since the GroHp circulates in many of the vector’s body parts, regardless of the presence of virus. An assay for direct binding might be possible with a Proximity ligation assay; however, it is beyond the scope of this project.

Future work should investigate additional molecular partners involved in virus–vector interactions, examine the role of GroHp under physiological conditions, and explore whether other endosymbiont-derived factors contribute to virus persistence and transmission in the beet leafhopper GroHp/GroEL.

## 5. Conclusions

This study investigates the interaction between the beet leafhopper endosymbiont-derived GroEL-homolog protein (SMBLH GroHp) and plant virus coat proteins. Although GroHp has been implicated in protecting and stabilizing virions in other insect–virus systems, our results demonstrate that SMBLH GroHp does not exhibit the strong binding specificity that has been reported in some other vector systems. Instead, SMBLH GroHp displayed relatively weak, and generally broad, interaction across multiple viral coat proteins, while *E. coli* GroEL demonstrated stronger and more generalized binding. These findings indicate that GroHp-CP interactions in the beet leafhopper system may be less selective than previously anticipated. Furthermore, GroHp-CP interactions alone are unlikely to fully account for vector specificity or efficient virus transmission in this system.

## Figures and Tables

**Figure 1:**
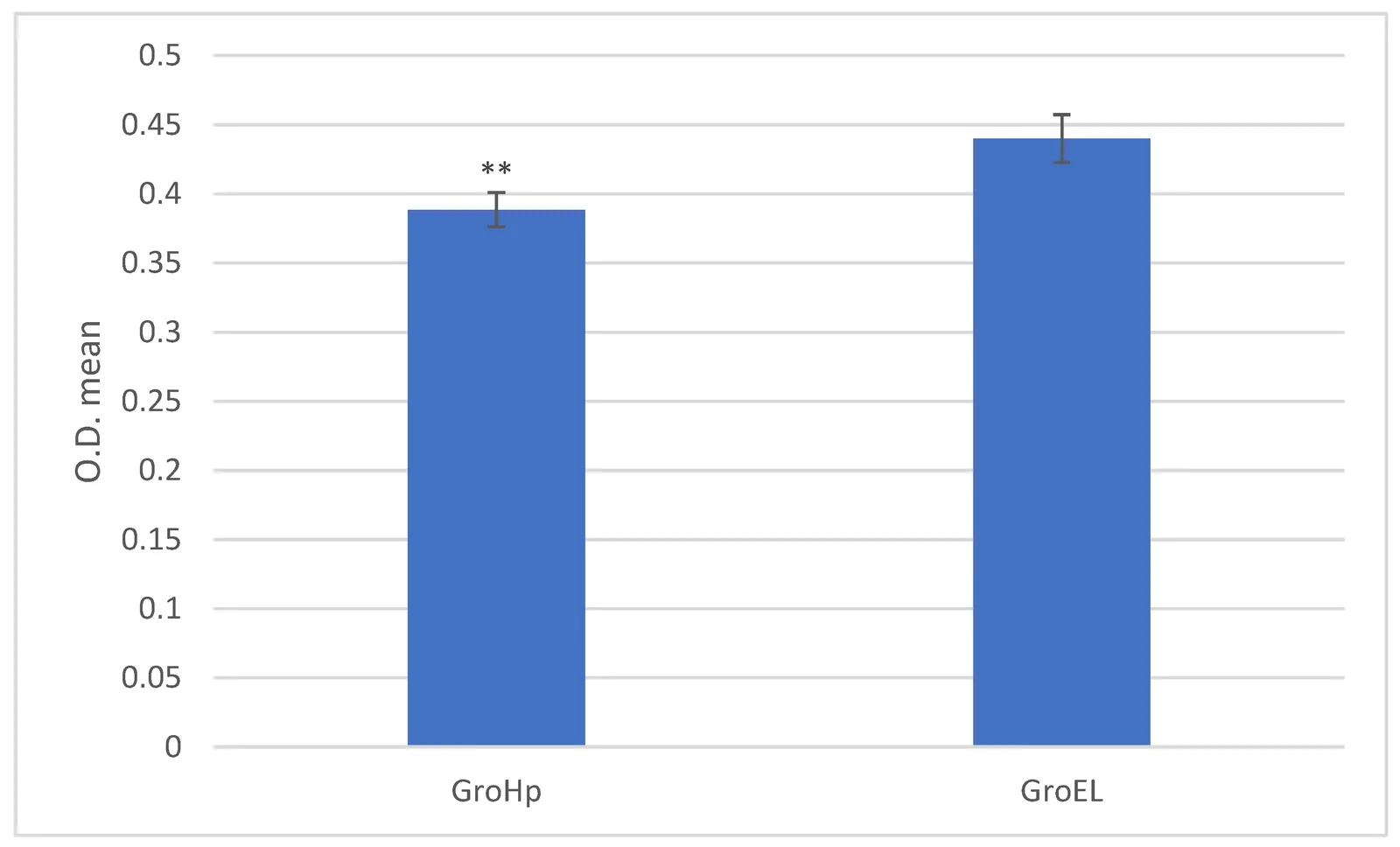
The cross-reactivity of the anti-*E. coli* GroEL antibody with SMBLH GroHp and *E. coli* GroEL proteins. An ELISA was conducted to determine if there is cross-reactivity between the anti-*E. coli* GroEL antibody and SMBLH GroHp and *E. coli* GroEL proteins. The results showed a significant difference in anti-*E. coli* GroEL antibody interaction with SMBLH GroHp and *E. coli* GroEL proteins. The anti-*E. coli* GroEL antibody has a greater affinity with GroEL than GroHp of about 12.43% more. (**) is *p*-value < 0.01.

**Figure 2:**
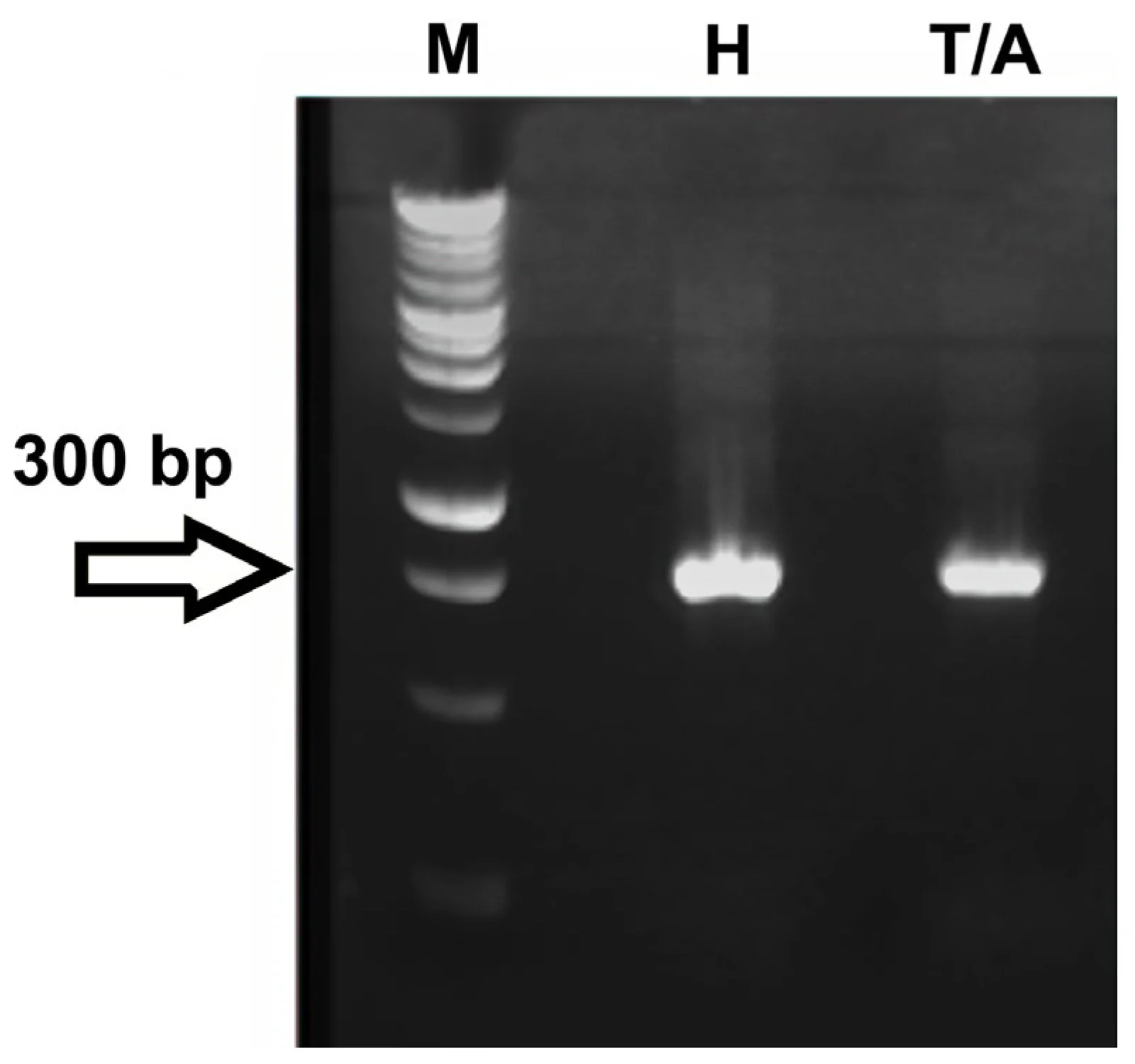
Detection of BCTV-Svr in both the head (H), and thorax and abdomen (T/H) of the BLH, using PCR. Both samples show the presence of curtovirus. The primers used were Rep primers, which yield a product of ~300 bp.

**Figure 3:**
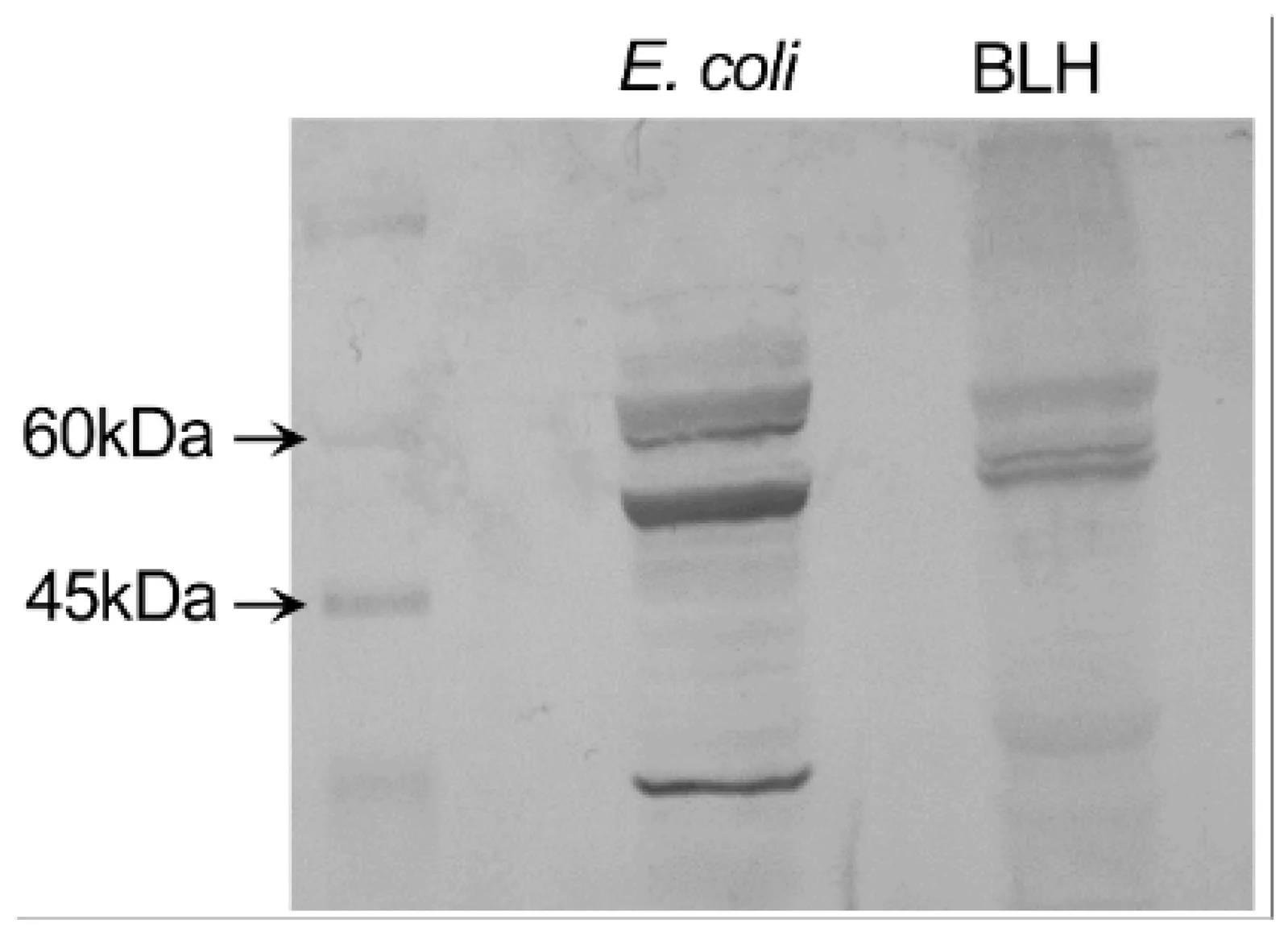
Immunodetection of BCTV-Svr interaction with GroEL/GroHp. Both crude proteins from heat-shock-induced *E. coli* and whole body BLH were incubated with BCTV-Svr-infected plant homogenate and then incubated with anti-BCTV-CP-antibody. The appearance of bands the same sizes of *E. coli* GroEL and SMBLH GroHp suggest that the GroEL/GroHp can capture the BCTV-Svr virus. The control was a lane of BCTV-Svr-infected plant. It showed no bands.

**Figure 4:**
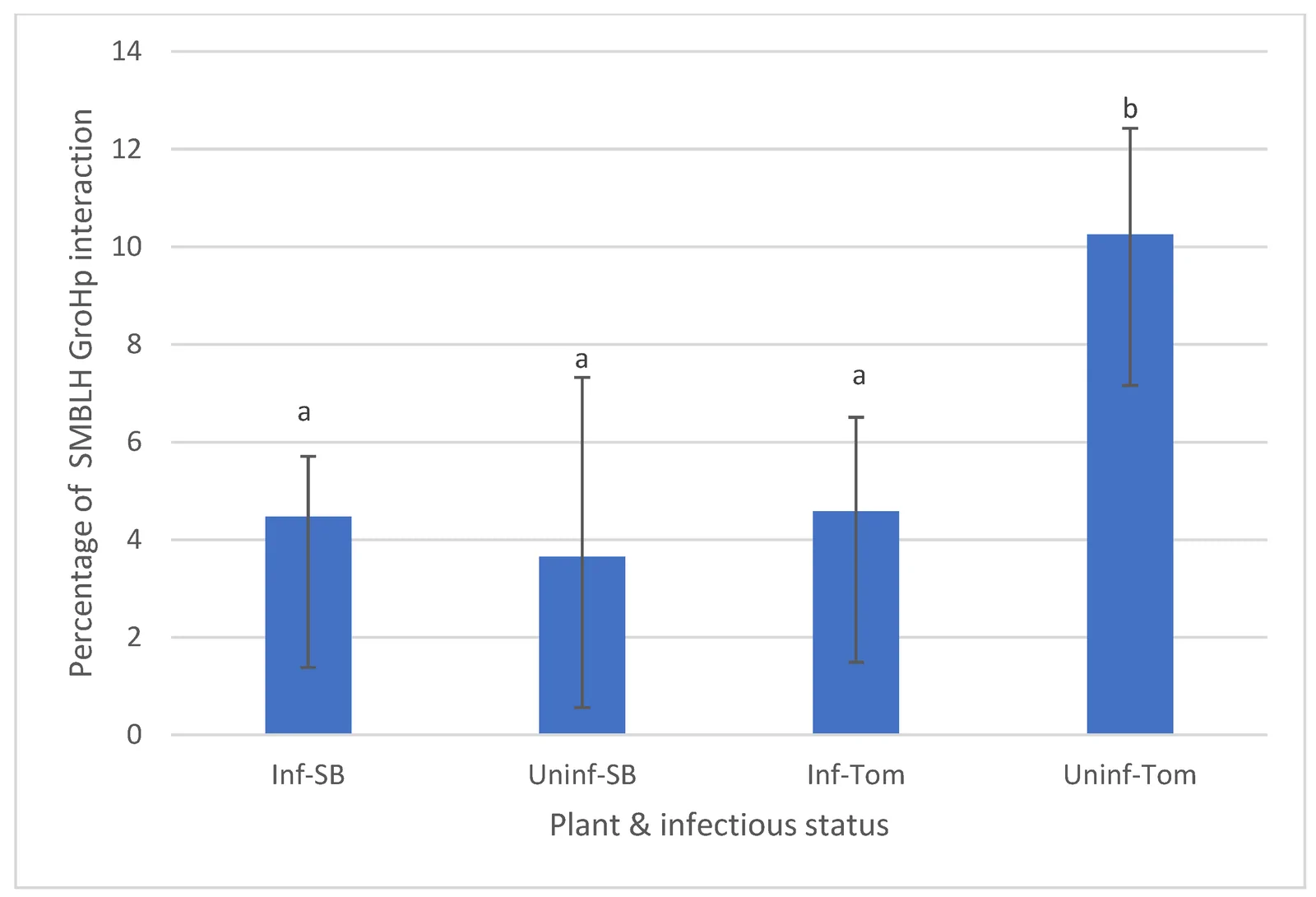
The percentage of GroHp interaction with homogenates of BCTV-infected and uninfected sugar beet, and TYLCV-infected and uninfected tomato plant. An ELISA was conducted to determine if SMBLH GroHp interacted with BCTV-infected and uninfected sugar beet and with TYLCV-infected and uninfected tomato plants. The graph shows the percentage on interaction. No significant difference exists between the GroHp interaction with plant homogenates from infected or uninfected sugar beet, and infected tomato (all labeled a). The only statistically significant difference can be seen with uninfected tomato plants (labeled b). Matching etters above a column indicate no significance difference, while distinct letters denote statistically significant variations between groups. The control was coating ELISA wells with purified GroHp, instead of plant homogenates, then adding GroHp protein. The O.D. readings were used to calculate the percentage of protein interactions.

**Figure 5:**
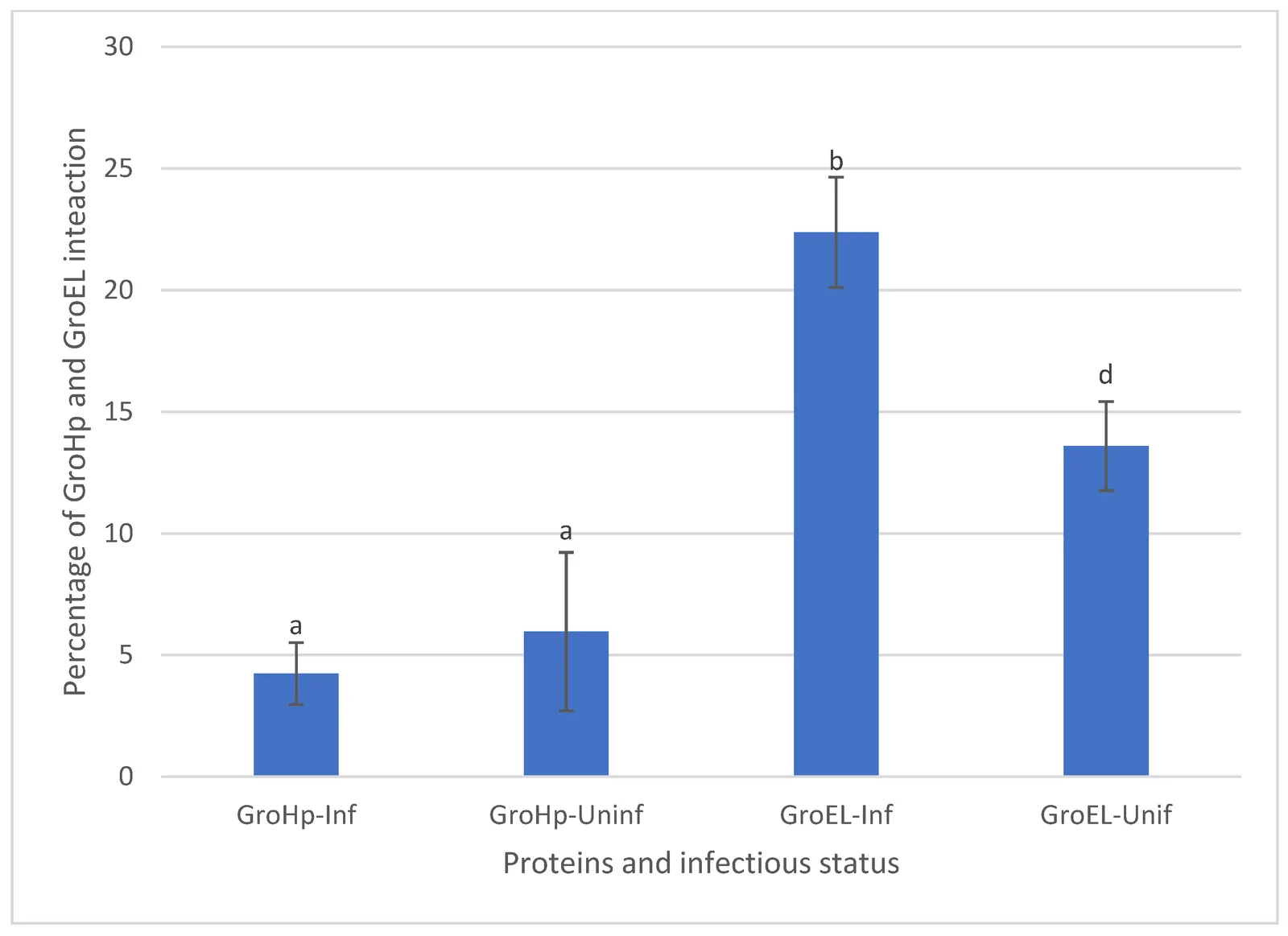
The percentage of GroHp vs. GroEL interaction with homogenates of BCTV-infected and uninfected sugar beet. An ELISA was conducted to determine if SMBLH GroHp and *E. coli* GroEL can interact with BCTV-infected and uninfected sugar beet. The results show that there was no significant interaction between infected (~4%) and uninfected (~6%) sugar beet with GroHp, but GroEL had significant interaction between *E. coli* and infected (~22%) and uninfected (~14%) sugar beet homogenate. The controls were coating ELISA wells with purified protein, instead of sugar beet homogenates, one row with GroHp and another with GroEL, then adding GroHp protein to the GroHp-coated wells and GroEL to the GroEL-coated wells. The O.D. readings were used to calculate the percentage of protein interactions. Matching letters above the columns indicate no significant difference, while distinct letters denote statistically significant variations between groups.

**Figure 6:**
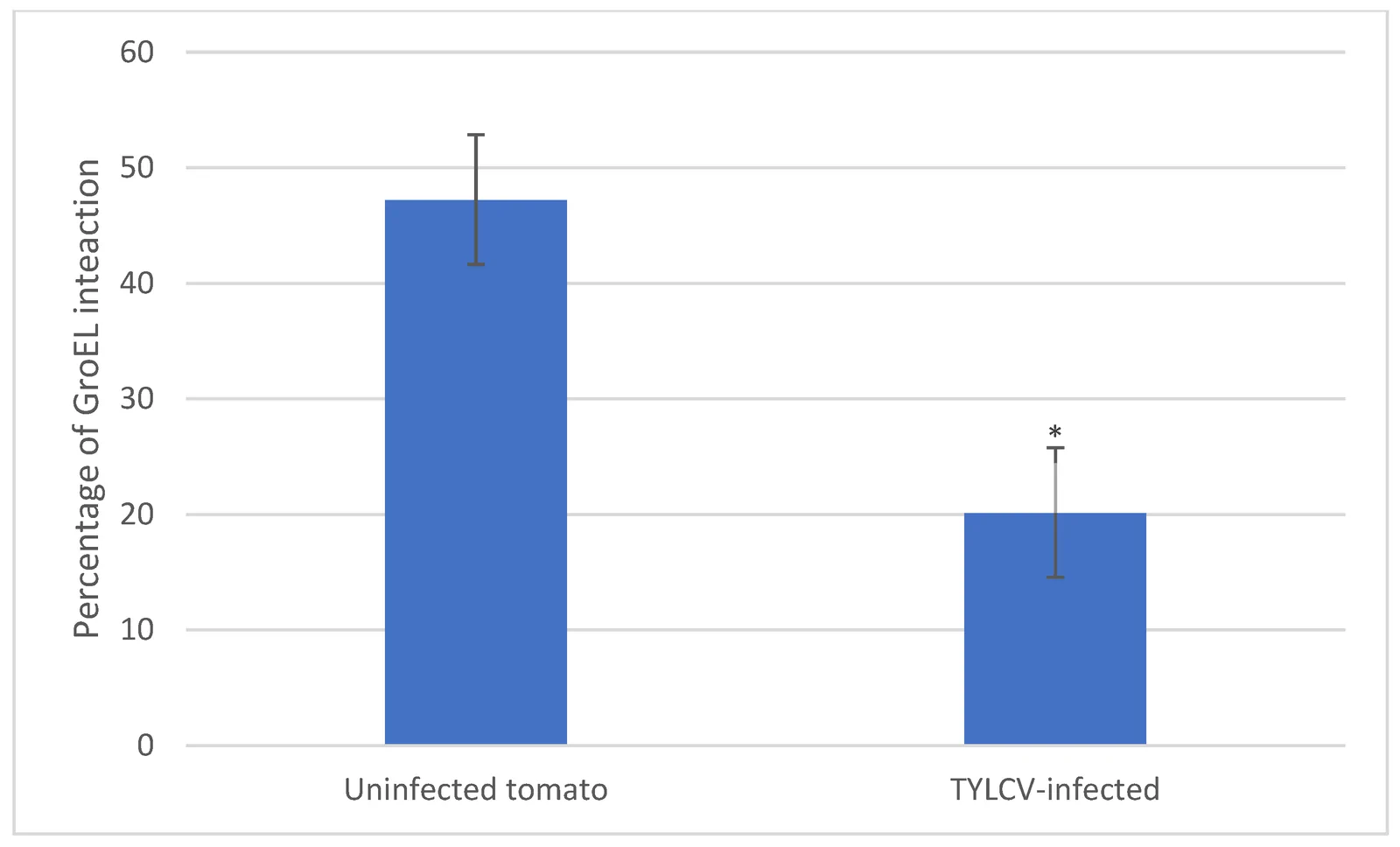
The percentage of GroEL interaction with TYLCV-infected and uninfected tomato homogenate. An ELISA was conducted to calculate the percentage of GroEL interaction with homogenates of uninfected tomato and TYLCV-infected tomato homogenate. There is a significant difference between both homogenates. GroEL has an interaction of ~48% with uninfected tomatoes which is more than twice as much as with TYLCV-infected tomatoes (~20%). (*) indicate *p* < 0.05.

**Figure 8:**
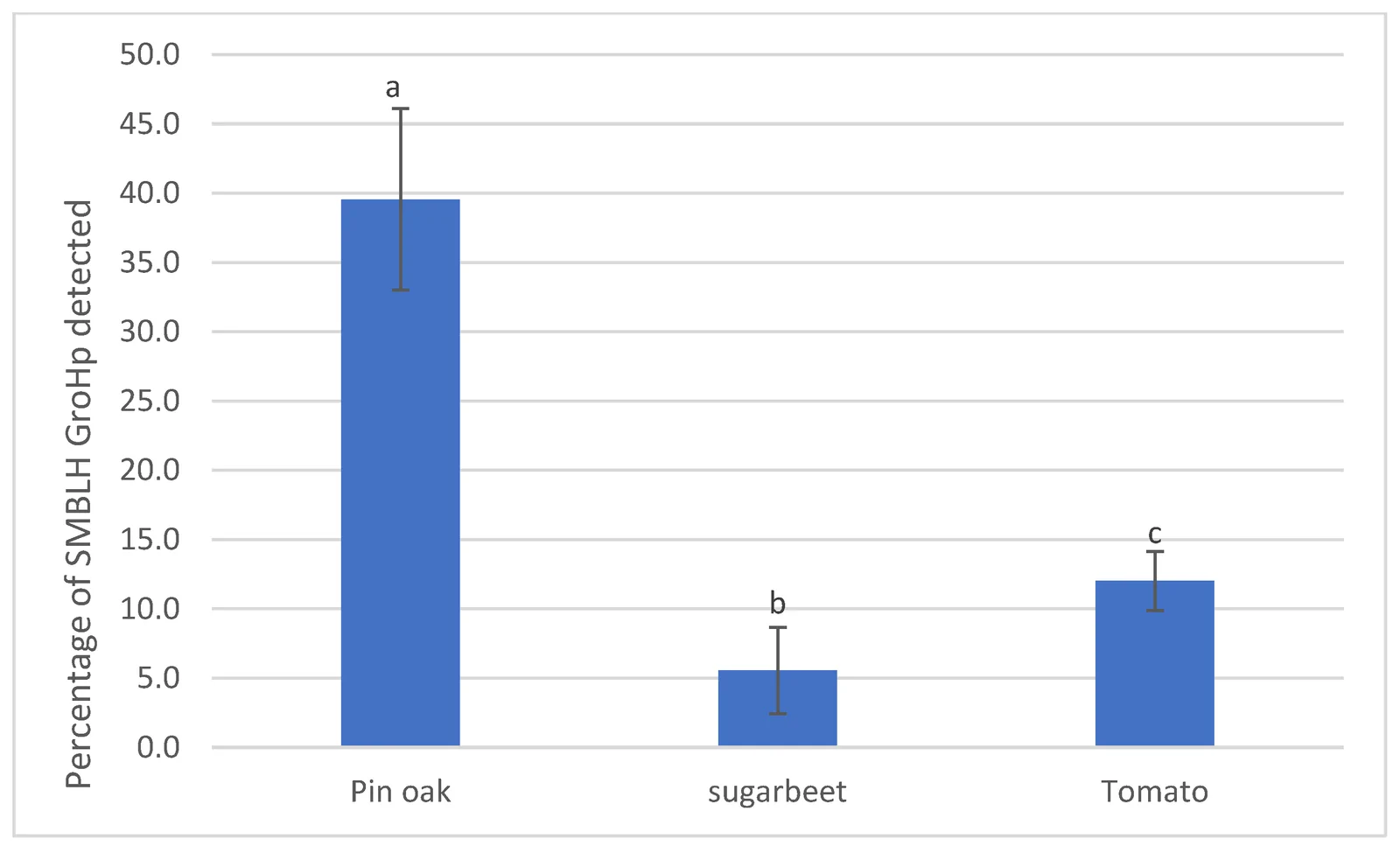
The percentage of GroHp interaction with homogenates of uninfected pin oak, sugar beet, and tomato plant. An ELISA was conducted to calculate the percentage of GroHp interaction with homogenates of uninfected pin oak, sugar beet, and tomato plant. All three homogenates showed significant interaction with GroHp, and all three were significantly different from each other. The greatest interaction was GroHp with pin oak at 39%, followed by that with tomato at 12%, then with sugar beet at 6%. Matching letters above a column indicate no significance difference, while distinct letters denote statistically significant variations between groups.

**Figure 9:**
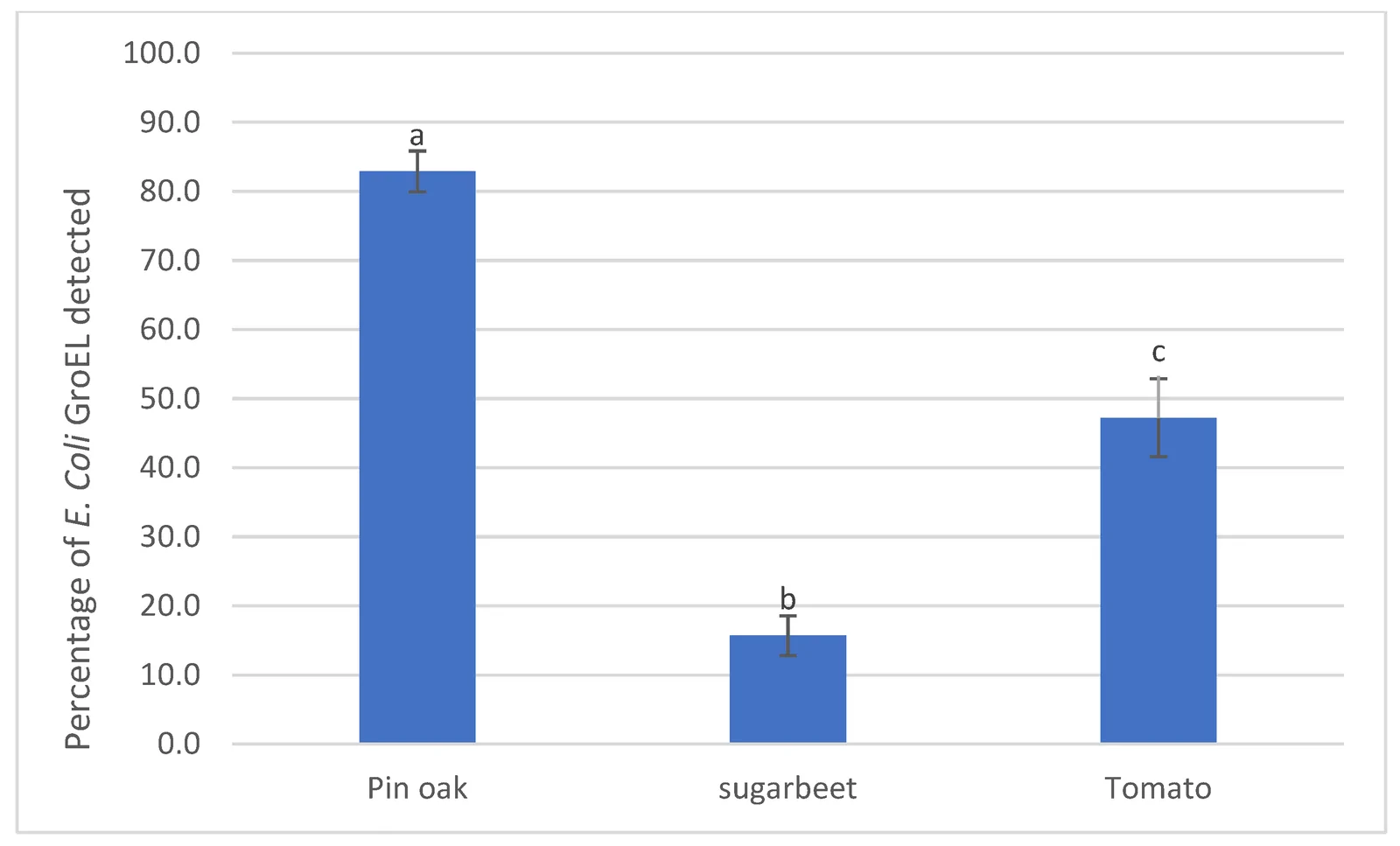
The percentage of GroEL interaction with homogenates of uninfected pin oak, sugar beet, and tomato plant. An ELISA was conducted to calculate the percentage of GroEL interaction with homogenates of uninfected pin oak, sugar beet, and tomato plant. All three homogenates showed significant interaction with GroEL, and all three were significantly different from each other. The greatest interaction was GroEL with pin oak at 83%, followed by that with tomato at 47%, then with sugar beet at 16%. Matching letters above a column indicate no significance difference, while distinct letters denote statistically significant variations between groups.

**Figure 10:**
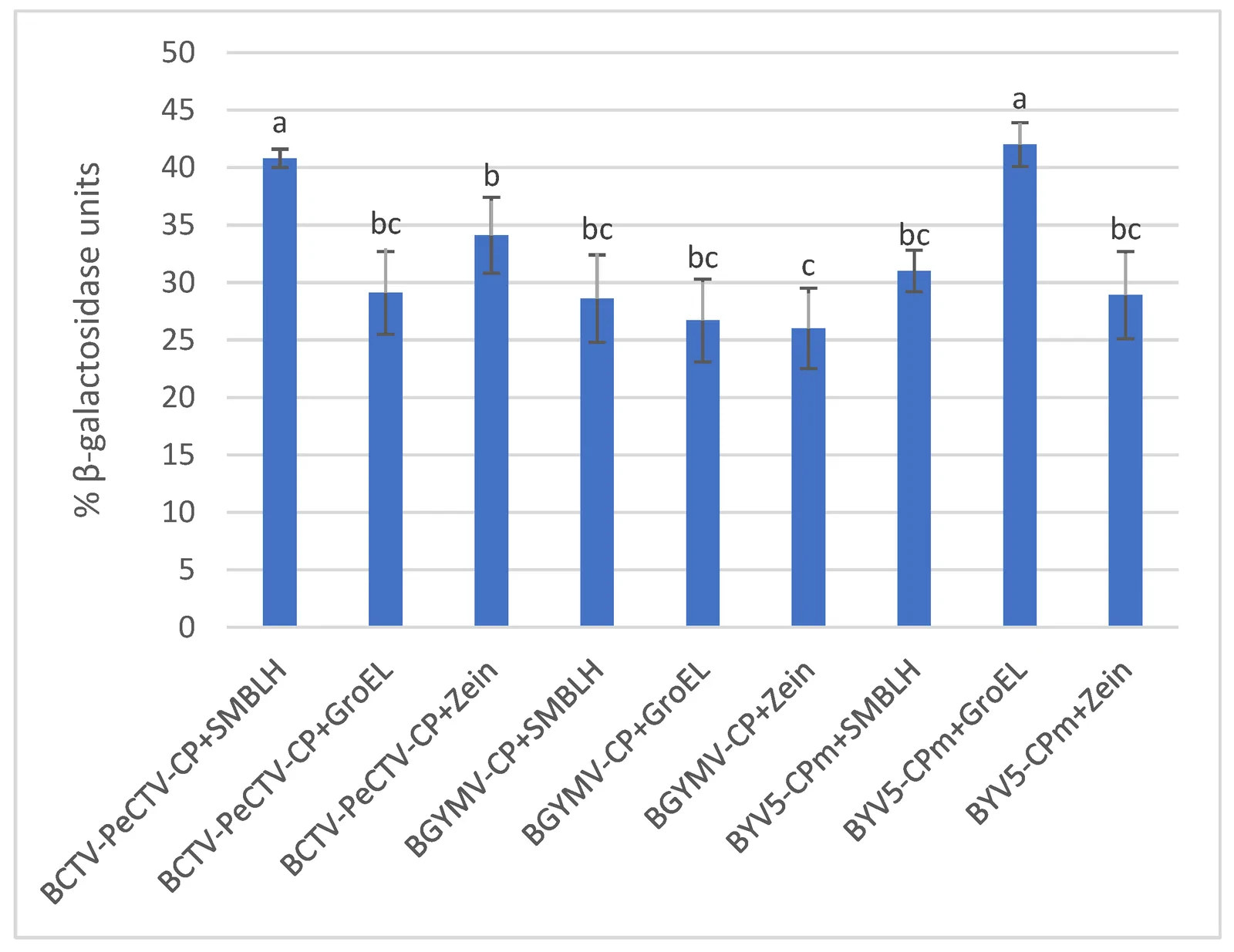
The percentage of β-galactosidase units produced by the BacterioMatch^TM^ system of protein–protein interactions. The letters ‘a, b, c’ represent groupings of statistical significance. The affinity of BCTV-PeCTV-CP to SMBLH GroHp is stronger than to *E. coli* GroEL, and zein proteins. The bars represent the standard error, and *p* < 0.05.

**Table 1:** Pairings and the results of co-transformation of the pBT and pTrg constructed fusion proteins. All fusion protein constructs exhibited colony growth after co-transformation into the reporter strain. The only pairings that failed to show colony growth were those in which at least one vector lacked a cloned gene (empty), as in all pairings with empty pTrg, and the pairing of empty pBT and empty pTrg.

pBT	pTrg	Colony Growth
BCTV-PeCTV-CP	SMBLH GroHp	+
	*E. coli* GroEL	+
	Zein	+
	Empty pTrg	−
BGYMV-CP	SMBLH GroHp	+
	*E. coli* GroEL	+
	Zein	+
	Empty pTrg	−
BYV5-CPm	SMBLH GroHP	+
	*E. coli* GroEL	+
	Zein	+
	Empty pTrg	−
Empty pBT	Empty pTrg	−
Gal 4	Gal 11P	+

**Table 2:** PCR-capture. The GroHp/GroEL starting dilution 10^−1^ (concentration 0.1 mg/mL). The PCR tubes were coated with serial dilutions of GroHp or GroEL from 10^−1^ to 10^−8^ (0.1 mg/mL to 0.01 ng/mL, respectively). The coated tubes were incubated with infected or noninfected cleared leaf homogenate, washed, and subjected to PCR using BCTV-PeCT, TYLCV, and TMV-CP-specific primers. (A) Represents capture by 10^−1^ to 10^−8^ dilutions of SMBLH GroHp-coated tubes. (B) Represents capture by 10^−1^ to 10^−8^ dilutions of E. coli GroEL PCR tubes. (C) Is negative control; tubes that were not coated. (D) Positive control, coating with extracted DNA from infected plants. (E) Coating the tubes with infected plant homogenate. A (+) indicate the presence of a capture, while (−) indicate no capture; N/A, not applied.

	(A) SMBLH GroHp 10^−1^–10^−8^ Dilution	(B) *E. coli* GroEL 10^−1^–10^−8^ Dilution	(C) Negative Control; Uncoated Tubes	(D) Positive Control. Infected-Plant-DNA-Coated Tubes	(E) Positive Control Infected-Plant-Homogenate-Coated Tubes
BCTV-PeCT-infected plant	+	+	−	+	+
TYLCV-infected plant	+	+	−	+	+
TMV-infected plant	+	+	−	+	+
Noninfected plant	−	−	−	−	N/A

**Table 3:** The β-galactosidase units for each group, of each trial of the pBT + pTrg cloned plasmids of the bacterial-two-hybrid assay. The pairings that showed no colony growth after co-transformation were not included in the results. The *µ* shows the average of the units.

	pTrg + pBT (Groups)	n	Avg. (*µ*)	%	Std. Error
1	BCTV-PeCTV-CP + SMBLH	7	54.5	40.8	0.8
2	BCTV-PeCTV-CP + GroEL	7	38.8	29.1	3.6
3	BCTV-PeCTV-CP + Zein	7	45.5	34.1	3.3
4	BGYMV-CP + SMBLH	7	38.2	28.6	3.8
5	BGYMV-CP + GroEL	7	35.6	26.7	3.6
6	BGYMV-CP + Zein	7	34.7	26	3.5
7	BYV5-CPm + SMBLH	7	41.3	31	1.8
8	BYV5-CPm + GroEL	7	56	42	1.9
9	BYV5-CPm + Zein	7	38.5	28.9	3.8
10	Gal11+Gal4/ + ve Control	7	133	100	9.8

## Data Availability

The original contributions presented in this study are included in the article/Supplementary Material. Further inquiries can be directed to the corresponding author.

## Supplementary Materials

The following supporting information can be downloaded at https://www.mdpi.com/article/10.3390/microorganisms14102176/s1, Figure S1. BLH head with arrows indicating the salivary glands. In A & B, the head was incubated with primary antibody (Anti-GroEl-Ab, for A, and Anti-BCTV-CP-Ab, for B). The heads were washed then incubated with a secondary fluorescent-Ab. A: BLH head with red fluorescence indicates the presence of GroHp. B: Red fluorescence showing the presence of BCTV. C: The control BHL head. The BLH head was not incubated with any primary antibody, but with only secondary antibody. It does not show red fluorescence; Figure S2. Western blot of BLH body parts, Head (H), thorax (T), and abdomen (A). The blot was incubated with Anti-*E. coli* GroEL-antibody. Protein bands of ~60kDa in the three body parts of BLH, as well as a faint mark from the beetle (Btl), can be seen; Figure S3. PCR-capture of BCTV-PeCT from infected plants. PCR tubes were coated with serial dilutions (10^−1^ to 10^−8^) of partially purified GroEL/GroHp (1 mg protein/ml), and then incubated with cleared leaf homogenate, washed, subjected to PCR using BCTV-PeCT, TYLCV, and TMV-CP specific primers. All tubes were incubated with infected plant homogenate in TBST. Two positive (Pos) controls were used. (1) for GroHp interaction with undiluted BCTV-PeCTV-infected plant homogenate. (2) for GroEL interaction with undiluted BCTV-PeCTV-infected plant homogenate. The negative control (Neg) was tubes coated with GroHp or GroEL but not incubated with any plant homogenate. (A) Represents capture by 10^−3^ to 10^−8^ dilutions of SMBLH GroHp and GroEL-coated tubes incubated with homogenates from TYLCV-infected plants. All concentrations-with both protein-showed interaction with the infected plant homogenate. (B) Represents capture by 10^−1^ to10^−8^ dilutions of SMBLH GroHp and GroEL-coated tubes incubated with homogenates from BCTV-PeCTV-infected plants. All concentrations-with both proteins-showed interaction with the infected plant homogenate. (C) Represents capture by 10^−2^ to10^−8^ dilutions of SMBLH GroHp and GroEL-coated tubes incubated with homogenates from TMV-infected plants. All concentrations-with both proteins-showed interaction with the infected plant homogenate; Table S1 Primers used for cloning into BacterioMatch^TM^ vectors. Underlined nucleotides indicate the restriction enzyme. Lowercase bases are not part of the gene. The antibiotic resistance is indicated by Ab^r^; Table S2: comparing the binding differences of anti-*E. coli* GroEL antibody with SMBLH GroHp and *E. coli* GroEL proteins. The results showed significant difference of how the antibody interacts with either protein; Table S3. The GroHp level was tested between viruliferous and aviruliferous BLH body parts. There were no significant differences between infected and uninfected Head, or thorax, or abdomen, furthermore, there was no significant differences in the level of GroHp between the the body parts for either infectious status; Table S4. ELISA detection of SMBLH GroHp and E. coli GroEL with Infected and Uninfected plants. GroHP does not favor interaction with any infected host plant, including sugarbeet. While GroEL shows better interaction when compared to GroHp. Both proteins had stronger interaction with uninfected plants compared to infected ones; Table S5. Comparing the level of GroHp between BCTV-infected sugar beet plants (Inf-SB), uninfected sugarbeets (Uninf-SB), TYLCV-infected tomato plants (Inf-Tom), and uninfected tomato plants (Uninf-Tom). The results showed there was no significant differences between the level of GroHp between infected and uninfected sugar beets, but a significant difference of the level of GroHp between infected and uninfected tomato plants and uninfected sugar beets vs uninfected tomatoes; Table S6. Comparing the level of SMBLH GroHp and *E. coli* GroEL in BCTV-infected and uninfected sugarbeet plants; Table S7. The results of ANOVA analysis. The F-calculated value > F-critical value. This indicates that at least one *µ* of one of the pairings is significantly different from the others.

## Abbreviations

The following abbreviations are used in this manuscript:

BLH	Beet leafhopper
GroHp	GroEL-homolog protein
CP	Coat protein
PCR	Polymerase chain reaction
