# Supplementary material for: Limited Specificity of GroEL-Homolog Interactions with Plant Virus Coat Proteins Challenges Models of Vector-Mediated Virus Transmission
Source: Microorganisms. 2026 Sep 27;14(10):2176. doi: 10.3390/microorganisms14102176 (PMC13707863; doi:10.3390/microorganisms14102176)
Supplement: Supplementary file 1 [file microorganisms-14-02176-s001.zip › Suplementary Figures.pdf]

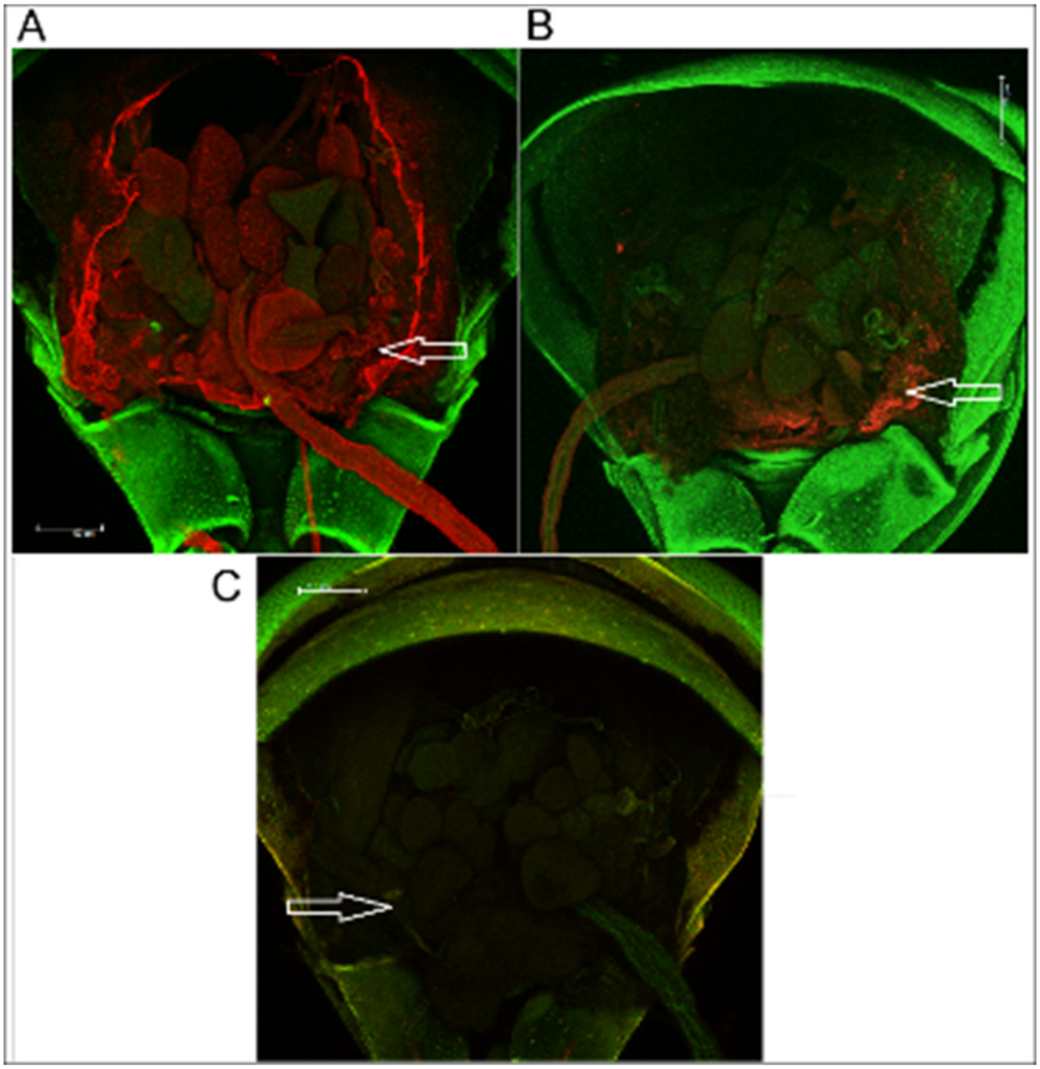

Supplementary Figure S1. BLH head with arrows indicating the salivary glands. In A & B, the head was incubated with primary antibody (Anti-GroEl-Ab, for A, and Anti-BCTV-CP-Ab, for B). The heads were washed then incubated with a secondary fluorescent-Ab. **A:** BLH head with red fluorescence indicates the presence of GroHp. **B:** Red fluorescence showing the presence of BCTV. **C**: The control BHL head. The BLH head was not incubated with any primary antibody, but with only secondary antibody. It does not show red fluorescence

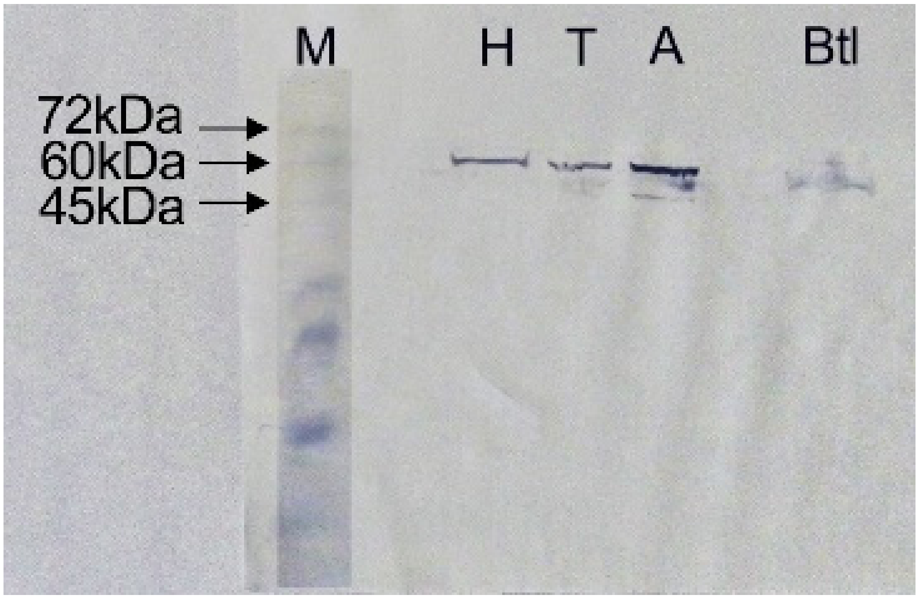

Figure S2. Western blot of BLH body parts, Head (H), thorax (T), and abdomen (A). The blot was incubated with Anti-*E. coli* GroEL-antibody. Protein bands of ~60kDa in the three body parts of BLH, as well as a faint mark from the beetle (Btl), can be seen.

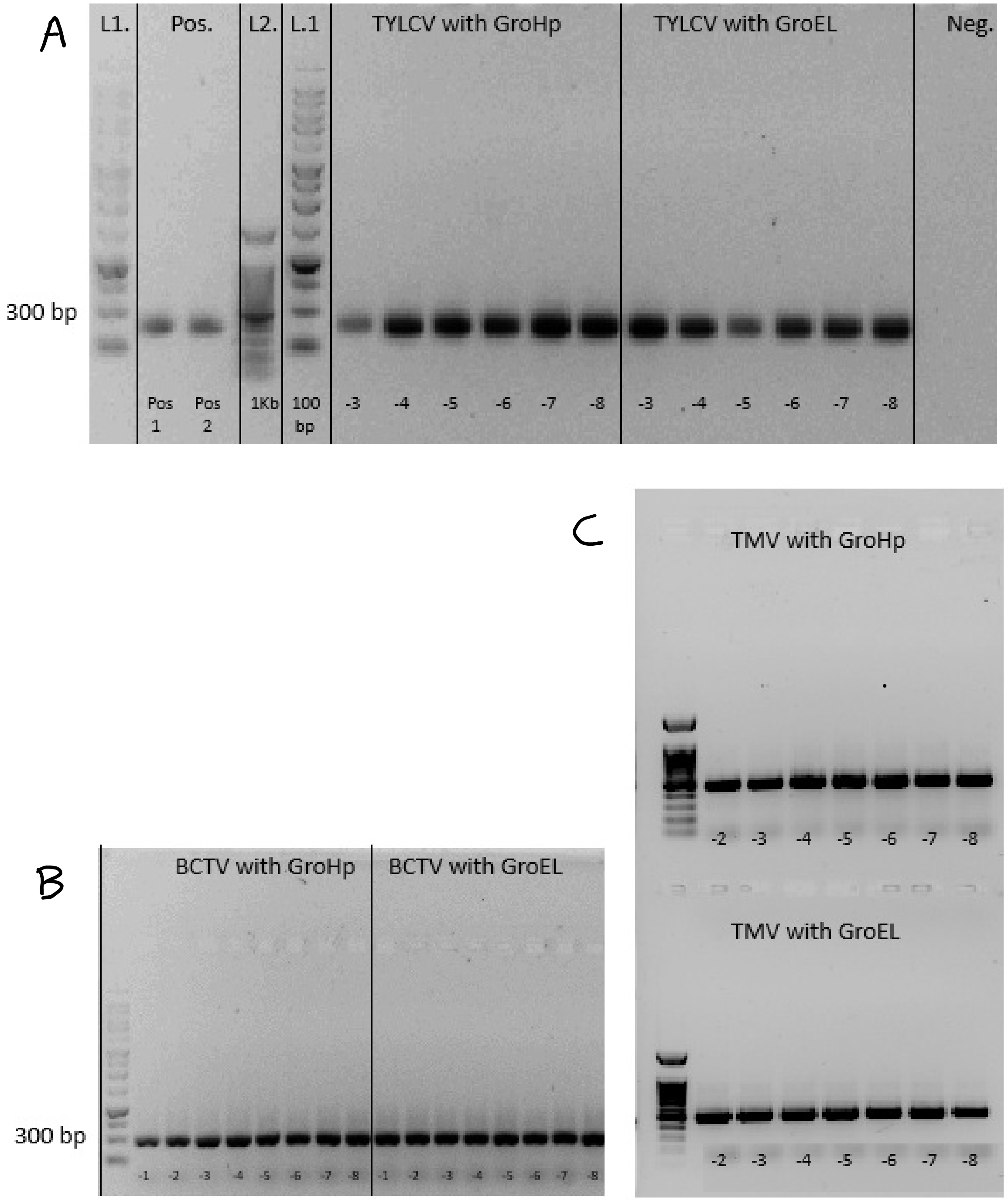

Figure S3. PCR-capture of BCTV-PeCT from infected plants. PCR tubes were coated with serial dilutions (10^{-1} to 10^{-8}) of partially purified GroEL/GroHp (1 mg protein/ml), and then incubated with cleared leaf homogenate, washed, subjected to PCR using BCTV-PeCT, TYLCV, and TMV-CP specific primers. All tubes were incubated with infected plant homogenate in TBST. Two positive (Pos) controls were used. (1) for GroHp interaction with undiluted BCTV-PeCTV-infected plant homogenate. (2) for GroEL interaction with undiluted BCTV-PeCTV-infected plant homogenate. The negative control (Neg) was tubes coated with GroHp or GroEL but not incubated with any plant homogenate. (A) Represents capture by 10^{-3} to10^{-8} dilutions of SMBLH GroHp and GroEL-coated tubes incubated with homogenates from TYLCV-infected plants. All concentrations-with both protein-showed interaction with the infected plant homogenate. (B) Represents capture by 10^{-1} to10^{-8} dilutions of SMBLH GroHp

and GroEL-coated tubes incubated with homogenates from BCTV-PeCTV-infected plants. All concentrations-with both proteins-showed interaction with the infected plant homogenate. (C) Represents capture by 10^{-2} to10^{-8} dilutions of SMBLH GroHp and GroEL-coated tubes incubated with homogenates from TMV-infected plants. All concentrations-with both proteins-showed interaction with the infected plant homogenate.
