# Supplementary material for: Limited Specificity of GroEL-Homolog Interactions with Plant Virus Coat Proteins Challenges Models of Vector-Mediated Virus Transmission
Source: Microorganisms. 2026 Sep 27;14(10):2176. doi: 10.3390/microorganisms14102176 (PMC13707863; doi:10.3390/microorganisms14102176)
Supplement: Supplementary file 1 [file microorganisms-14-02176-s001.zip › Supplementary Tables.pdf]

Table S1 Primers used for cloning into BacterioMatch™ vectors. Underlined nucleotides indicate the restriction enzyme. Lowercase bases are not part of the gene. The antibiotic resistance is indicated by Ab^r

Gene/Primer	Sequence	Annealing temp	Strategene vector/ Ab^r
E. coli groel			
EcoliNot_F	aaagGCGGCCGCAATGGCAGCTAAAGACG	57 °C	pBT/Cam^r
EcoliXhoIR	AA CTCGAGGCAGGGCAATTA		
**SMBLH *groel***			
SulNot_CT_F1	aaagGCGGCCGCAATGGCAAAAAATATTCA	57 °C	pBT/Cam^r
XhoISulgen_R	CTGCTGCTCGAGCGAAGATTTTCCTTTTT		
**Curtovirus CP (BCTV-PeCTV-CP)**			
Pectv_Bam_F'	aaatgaGGATCCATGTCGAGATTTACAAAAGGT A	57 °C	pTrg/Tet^r
XhoPeCTVBV3R	AACTCGAGCAATCTTCAATCTCG		
**Begomovirus CP (BGYMV-CP)**			
BegoBamF′	aaGGATCCATGTATGCCCATTTTACG	55 °C	pTrg/Tet^r
XhoIBego_CP_R	CTCGAGTGAGAATCATGAAATAAAATA		
**Closterovirus CPm (BYV5-CPm)**			
BYV5-NotIF	GCGGCCGCA ATGTTGGCGCCCGAAGGCAG	65.5 °C	pTrg/Tet^r
BYVORF 5 R	CTCGAGTTAACTAAGACGACCGAGGTCGAAAAG		

Table S2: comparing the binding differences of anti-*E. coli* GroEL antibody with SMBLH GroHp and *E. coli* GroEL proteins. The results showed significant difference of how the antibody interacts with either protein.

ANOVA

Source of Variation	*SS*	*df*	*MS*	*F*	*P-value*	*F crit*
Between Groups	0.021218	1	0.021218	95.18355	8.1E-11	4.170877
Within Groups	0.006688	30	0.000223			
Total	0.027906	31				
St Div of mean	0.0123	0.0172				

Table S3. The GroHp level was tested between viruliferous and aviruliferous BLH body parts. There were no significant differences between infected and uninfected Head, or thorax, or abdomen, furthermore, there was no significant differences in the level of GroHp between the the body parts for either infectious status.

Anova: Two-Factor With Replication

Source of Variation	*P-value*
Comparing the three BLH body parts to one another	0.128744
Comparing differences within viruliferous and aviruliferous status	0.42471
Comparing the interaction of each body part with each viruliferous and aviruliferous status	0.385395

Table S4. ELISA detection of SMBLH GroHp and E. coli GroEL with Infected and Uninfected plants. GroHP does not favor interaction with any infected host plant, including sugarbeet. While GroEL shows better interaction when compared to GroHp. Both proteins had stronger interaction with uninfected plants compared to infected ones.

Percentage of SMBLH GroHp and *E.coli* GroEL interact with different host/non host plants

	GroHp	GroEL		GroHp	GroEL
TMV-infected tobacco	~4.5%	49%			
BCTV-infected sugar beet	~4.5%	~22%	Uninfected sugar beet	~6%	~14%
TYLCV-infected tomato	~4.3%	~20%	Uninfected tomato	~12%	~48%
			Pin oak	~39%	~82%

Table S5. Comparing the level of GroHp between BCTV-infected sugar beet plants (Inf-SB), uninfected sugarbeets (Uninf-SB), TYLCV-infected tomato plants (Inf-Tom), and uninfected tomato plants (Uninf-Tom). The results showed there was no significant differences between the level of GroHp between infected and uninfected sugar beets, but a significant difference of the level of GroHp between infected and uninfected tomato plants and uninfected sugar beets vs uninfected tomatoes.

**Treatment pairs**	**Tukey HSD p-value**	**Inference**
Inf-SB vs. Uninf-SB	0.773125	Insignificant
Inf-SB vs Inf-Tom	0.899995	Insignificant
Inf-SB vs Uninf-Tom	0.001005	**p<0.01
Uninf-SB vs. Inf-Tom	0.730534	Insignificant
Uninf-SB vs. Uninf-Tom	0.001005	**p<0.01
Inf-Tom vs. Uninf-Tom	0.001005	**p<0.01

Table S6. Comparing the level of SMBLH GroHp and *E. coli* GroEL in BCTV-infected and uninfected sugarbeet plants.

Treatment pairs	Tukey HSD p-value	Inference
GroHp in Inf vs. GroHp in Uninf	0.447651	Insignificant
GroHpin Inf vs GroEL in Inf	0.001005	**p<0.01
GroHp in Inf vs. GroEL in Uninf	0.001005	**p<0.01
GroHp in Uninf vs. GroEL in Inf	0.001005	**p<0.01
GroHp in Uninf vs. GroEL in Uninf	0.001005	**p<0.01
GroEL in Iinf vs. GroEL in Uninf	0.001005	**p<0.01

Table S7. The results of ANOVA analysis. The F-calculated value>F-critical value. This indicates that at least one μ of one of the pairings is significantly different from the others.

Source of Variation	*SS*	*df*	*MS*	*F*	*P-value*	*F crit*
**Between Groups**	47416	9	5268.39653	39.45824	1.12E-19	2.073351
**Within Groups**	6675.9	50	133.51829			
